# Cluster-assembled zirconia substrates promote long-term differentiation and functioning of human islets of Langerhans

**DOI:** 10.1038/s41598-018-28019-3

**Published:** 2018-07-02

**Authors:** Alessandra Galli, Elisa Maffioli, Elisa Sogne, Stefania Moretti, Eliana Sara Di Cairano, Armando Negri, Simona Nonnis, Giuseppe Danilo Norata, Fabrizia Bonacina, Francesca Borghi, Alessandro Podestà, Federico Bertuzzi, Paolo Milani, Cristina Lenardi, Gabriella Tedeschi, Carla Perego

**Affiliations:** 1 Dipartimento di Scienze Farmacologiche e Biomolecolari, Università degli Studi di Milano, via Trentacoste 2, 20134 Milan, Italy; 2 Dipartimento di Medicina Veterinaria, Università degli Studi di Milano, via Celoria 10, 20133 Milan, Italy; 3 Fondazione Filarete, v.le Ortles 22/4, 20139 Milan, Italy; 4 CIMAINA and Dipartimento di Fisica, Università degli Studi di Milano, via Celoria 16, 20133 Milan, Italy; 5 Niguarda Hospital, Milan, Italy; 6 Present Address: Biological and Environmental Science and Engineering Division, KAUST, Jeddah, Saudi Arabia

## Abstract

*Ex vivo* expansion and differentiation of human pancreatic β-cell are enabling steps of paramount importance for accelerating the development of therapies for diabetes. The success of regenerative strategies depends on their ability to reproduce the chemical and biophysical properties of the microenvironment in which β-cells develop, proliferate and function. In this paper we focus on the biophysical properties of the extracellular environment and exploit the cluster-assembled zirconia substrates with tailored roughness to mimic the nanotopography of the extracellular matrix. We demonstrate that β-cells can perceive nanoscale features of the substrate and can convert these stimuli into mechanotransductive processes which promote long-term *in vitro* human islet culture, thus preserving β-cell differentiation and function. Proteomic and quantitative immunofluorescence analyses demonstrate that the process is driven by nanoscale topography, via remodelling of the actin cytoskeleton and nuclear architecture. These modifications activate a transcriptional program which stimulates an adaptive metabolic glucose response. Engineered cluster-assembled substrates coupled with proteomic approaches may provide a useful strategy for identifying novel molecular targets for treating diabetes mellitus and for enhancing tissue engineering in order to improve the efficacy of islet cell transplantation therapies.

## Introduction

Diabetes mellitus (DM), primarily defined as a chronic hyperglycemia, is one of the most common and serious metabolic disorders which affected 382 million people worldwide in 2013 and is expected to afflict 592 million by 2035 (World Health Organization)^1^. Progressive β-cell dysfunction, dedifferentiation and death and the corresponding decrease in insulin production are the major components of all forms of diabetes.

β-cell replacement and/or regenerative strategies appear to be useful for long-term glucose control and preventing diabetes compliances. The limited availability of organ donors and/or the low viability of transplanted islets to immunosuppressive treatments has hindered the wide application of replacement therapies^2^. Regenerative strategies are still under development mainly due to our partial understanding of the signaling pathways controlling human β-cell replication and differentiation^3^.

Several strategies have been proposed for finding alternative sources of insulin-producing cells, including engineered human β-cells, human embryonic stem cells (hESCs) and human induced pluripotent stem cells (iPSCs)^4–6^. Recently developed protocols have greatly improved the glucose responsiveness of insulin-secreting cells generated *in vitro* from human pluripotent stem cells^7^, yet safety is still a major concern for any hESC or iPSC technology-based regenerative therapy. Organoids from adult pancreas and reprogramming of pancreatic epithelial cells (duct, acinar, or α-cells) into β-cells represent attractive alternatives to stem cells^8–11^. Translation of such capacity to human cells has yet to be achieved. Expansion of adult β-cells remains a promising strategy but it requires complex dedifferentiation and redifferentiation processes^12,13^.

Mature human β-cells are highly differentiated and specialized cells and *ex vivo* proliferation seldom occurs. Furthermore, in 2D cultures they progressively down-regulate insulin production, enzymes for insulin processing, lose glucose responsiveness and may undergo a dedifferentiation process toward an immature endocrine phenotype^14^ or die by apoptosis^15^. It is believed that the same processes occur in T2D^16^. Therefore, it is essential to identify the core mechanism controlling β-cell fate and function in order to increase β-cell mass and maintain the mature cell phenotype.

Like other tissues, β-cell behavior is strongly influenced by cell-cell and cell-matrix interactions. Adhesion between β-cells (promoted by E-cadherins and connexins) controls basal and stimulated insulin release^17,18^. Interactions with other insular cells, mediated by paracrine signals, shape β-cell fate and modulate the insulin secretion^19^. In mature, intact islets, endocrine cell proliferation and survival are strictly regulated by extracellular matrix (ECM) interactions^20–22^. Almost all major ECM molecules have been identified in pancreatic islets and most of them have been associated with specific biological processes. For example in human islets, collagen and fibronectin promote β-cell survival; laminins control β-cell differentiation and insulin secretion^23^. ECM proteins signal through membrane associated integrin and non-integrin receptors which sense modifications in the ECM composition and influence cell behavior through a complex intracellular signaling cascade^23^. Findings derived from these studies led to the development of 2D and 3D culture systems based on extracellular matrix components or biomimetic peptides which greatly enhanced β-cell survival and differentiation *in vitro*^24,25^.

In the last few years it has become clear that not only chemical, but also physical properties of the extracellular environment have a profound influence on cell behavior and differentiation and indeed a combination of micro- and nano-scale chemical and physical extracellular signals seems to regulate cell functions^26,27^. In their natural environment cells interact with extracellular matrix components, which are structured at nanometer scale. Accordingly, cells respond to nanoscale features when grown on nanostructured substrates and nanoscale topography has been found to influence cell adhesion and proliferation^28^. The relevance of topographic cues to cell responses is therefore unquestionable.

Nanostructured surfaces with complex morphology mimicking that of the ECM can be obtained by assembling clusters using Supersonic Cluster Beam Deposition (SCBD)^29,30^. The cluster-assembled surfaces are characterized by random nanoscale roughness that can be carefully controlled and can range from a few nanometers up to tens of nanometers^31^. The essential functions of cells grown on transition metal oxide nanostructured surfaces fabricated by SCBD, such as titania and zirconia, are strongly affected by nanoscale features. For example the nanoscale substrate suffices to promote differentiative behavior (neuritogenesis, synaptogenesis and maturation) of neuronal cells by mechanotransductive events^29,32,33^. The nanotopography of cluster-assembled surface also strongly affects the absorption of proteins and the IsoElectric Point^34^.

The mechanosensing ability of beta cells has been recognized, however its underlying mechanism remains largely unexplored. In this study, we fabricated clusters-assembled zirconia substrates with controlled morphological properties at the nanoscale and we demonstrated that these substrates promote the differentiation and function of human islets in long term *in vitro* islet cultures. Using a proteomic approach we characterized the molecular mechanisms involved in the ability of islets to transduce the topographical cues within a program which preserves β-cell survival and function.

## Results

### Structural characterization of zirconia substrates

Cluster-assembled thin films with different nanoscale roughness (ns-ZrO_x_) were grown on glass cover slides by depositing a seeded supersonic beam of ZrO_x_^35^ clusters, thus producing cubic zirconia films with tunable and stable nanoscale morphology against thermal annealing. Standard zirconia films were grown by atom assembling with an electron beam evaporator and were used as a control^29,35^.

**Figure 1:**
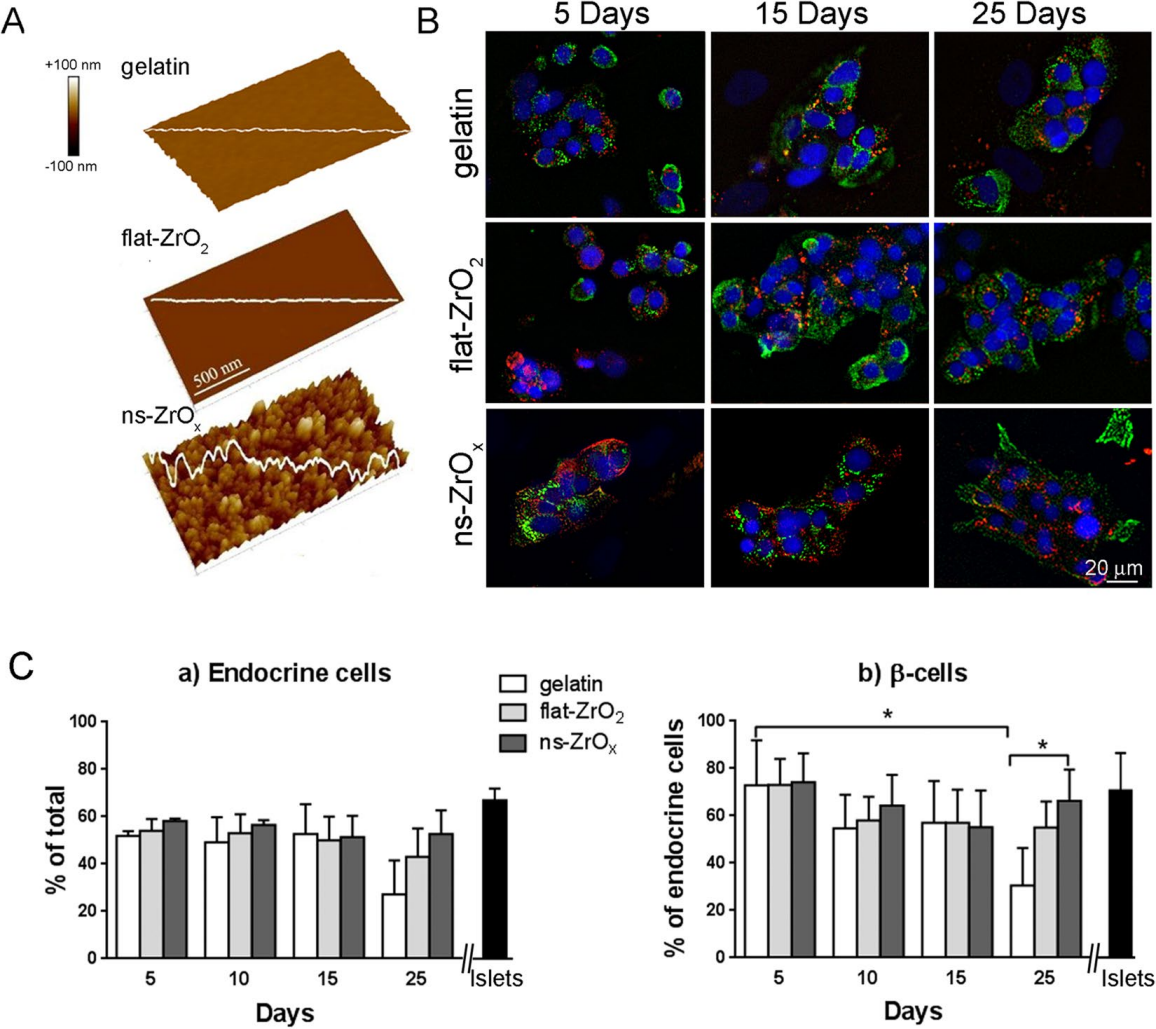
Nanostructured zirconia substrates promote long term β-cell survival. (**A**) Three-dimensional views of AFM topographic maps of substrates. Representative topographic profiles are superimposed over maps (white lines) in gelatin, flat-ZrO_2_ and cluster-assembled ns-ZrO_x_ (rms 15 nm). (**B**) Representative confocal images of human islets grown on different substrates for 5, 10 or 25 days. The cells were triple stained with anti-insulin (red), anti-chromogranin A (green) antibodies or DAPI (blue). The yellow-orange staining indicates colocalization between chromogranin A and insulin. Bar: 20 μm. (**C**) *Quantification of endocrine cells density*. Chromogranin A-positive endocrine cells are expressed as percentage of total DAPI-positive cells and are the mean ± SE of five independent experiments, performed in duplicate. The black bar reports the percentage of endocrine cells in freshly isolated islets. *Quantification of β-cell density*. Insulin-positive β-cells are expressed as percentage of total endocrine cells (chromogranin A-positive cells) and are the mean ± SE of five independent experiments, performed in duplicate. The black bar reports the percentage of β-cells in freshly isolated islets. (*p < 0.05, ns-ZrO_x_ *vs* gelatin).

Figure 1A shows three-dimensional views of AFM topographic maps of gelatin, flat zirconia film (flat-ZrO_2_) and cluster assembled zirconia films with rms roughness of 15 ± 0.6 nm (15-ns-ZrO_x_). The cluster-assembled films have a granular and nanoporous structure when compared with flat-ZrO_2_ (rms roughness 0.043 ± 0.010). The gelatin film surface was characterized by irregularities at nanoscale level due to protein clusters (Suppl Fig. S1A), but the rms roughness was less than 1 nm^36^. The surface profiles of ns-ZrO_x_ films are characterized by randomly distributed asperities at nanoscale resulting from the ballistic deposition regime typical of cluster assembling with SCBD^31^. The size and the spatial organization of these features resemble the structure of the ECM. This is quantitatively estimated by the specific area, calculated as the ratio of surface area to the projected area). The average value of the specific area was 1.008 ± 0.001 for flat samples which greatly increased up to 1.54 ± 0.03 (rms roughness 15 nm) and 1.66 ± 0.08 (rms roughness 20 nm) for the nanostructured samples. The ns-ZrO_x_ films were characterized by the BET method thus obtaining a specific surface area of approximately 300 m^2^/g and nanosized pores in the range of 10–50 nm.

### Nanostructured zirconia promotes long term endocrine cell survival

In order to test the role of nanoscale morphology of ns-ZrO_x_ in promoting β-cell viability and function *in vitro*, human islets were dispersed by a short trypsinization and cultured on flat-ZrO_2,_ 15-ns-ZrO_x_ and 20-ns-ZrO_x_ for up to 30 days. Gelatin coated glass coverslips (gelatin) were used as a control.

Within 24 h all of the islet cells attached to the different substrates with equal efficiency except for 20-ns-ZrO_x_. Phase contrast optical images of islets maintained in culture medium for 20 days showed the presence of islet-like cell clusters on ns-ZrO_x_ (Suppl. Fig S1B). A similar morphology was detected on gelatin and flat-ZrO_2_ but the central ‘core’ of the aggregated cells was surrounded peripherally by a migrating population of elongated cells. The cell density was similar on the different substrates except for 20-ns-ZrO_x_ where it was lower, probably due to inefficient cell adhesion observed over the 24 hours. For this reason 20-ns-ZrO_x_ was excluded from further analyses. The total protein content analysis confirmed these findings (Suppl. Fig. S1C).

In order to characterize the different cell populations and determine whether long term cultures on zirconia substrates preserve β-cell viability and differentiation, the percentage of endocrine β-cells in the various groups was evaluated by immunofluorescence at different stages after plating (Fig. 1B). The percentage of chromogranine-positive endocrine cells and insulin-positive β-cells grown on different substrates were similar at five days of culture (Fig. 1C). However, in long-term cultures (more than 20 days) although a similar fraction of endocrine cells were observed on the different substrates (with a lower trend on gelatin), a significantly higher percentage of β-cells was detected on ns-ZrO_x_ than on control substrates (41 ± 15% increase, p < 0.05; Fig. 1C). A similar trend was observed on flat-ZrO_2_.

Triple immunofluorescence staining with hormones showed the presence of mono-hormonal insulin-, glucagon- and somatostatin-positive endocrine cells in similar proportions on the different substrates after five days in culture (Suppl. Fig. S1D). This organization was only retained in long-term cultures in islets grown on ns-ZrO_x_; conversely numerous glucagon-expressing cells were detected on the control substrates. No bi-hormonal expressing cells were observed.

During the time course of the culture, the overgrowth of elongated fibroblast-like cells was detected on gelatin and flat-ZrO_2_ substrates. Staining with α-smooth muscle actin (α-SMA), a marker of myofibroblasts and mesenchymal cells, confirmed the “non endocrine” nature of these cells and showed that insulin-positive cells were completely surrounded by α-SMA-positive elongated cells on gelatin and flat-ZrO_2_ substrates for 20 days (Suppl. Fig. S1E). Conversely, a very small number of α-SMA-positive cells were detected around several insulin-positive cells that preserve their organization in islet-like clusters on ns-ZrO_x_ substrates. These α-SMA-positive cells may represent dedifferentiated β-cells or proliferating fibroblast/mesenchymal cells originally associated with the islets^14^.

Taken together, these data suggest that ns-ZrO_x_ is a promising substrate for long-term *in vitro* human islet culture, because it maintains β-cell survival and differentiation, preserves the cellular organization of intact islets and prevents overgrowth of fibroblast-like cells.

### Quantitative proteomic analyses reveal that ns-ZrO_x_ promotes β-cell survival and differentiation

In order to assess the molecular mechanisms responsible for the ability of ns-ZrO_x_ to maintain β-cell fate *in vitro*, the islets grown on different substrates for 20 days were collected, lysed and processed by a shotgun proteomic approach using label-free quantification, following the workflow briefly described in Suppl. Fig. S2. As shown in the Venn diagram reported in Suppl Fig. S3A, the analysis identified 49, 64 and 65 proteins exclusively expressed in gelatin, ns-ZrO_x_ and flat-ZrO_2_, respectively, and 1379 proteins present under all conditions of which 97 were differentially expressed (Anova test FDR 0.05) (Table 1). The bioinformatic analyses carried out on these proteins suggest that the main difference between the proteome of cells grown under the three different conditions is related to cytoskeleton, modulation of morphology and enzyme activity (Fig. S3B).

**Table 1:** Proteins differentially expressed in ns-ZrO_x_, gelatin and flat-ZrO_2_.

Protein IDs	Protein names	Gene names
A0A024R4E5	Vigilin	HDLBP
P08123	Collagen alpha-2(I) chain	COL1A2
Q96JY6	PDZ and LIM domain protein 2	PDLIM2
O00622	Protein CYR61	CYR61
A0A087X0x3	Heterogeneous nuclear ribonucleoprotein M	HNRNPM
Q9BUJ2	Heterogeneous nuclear ribonucleoprotein U-like protein 1	HNRNPUL1
A0A0A0MSS8	Aldo-keto reductase family 1 member C3	AKR1C3
A0A0A0MTS2	Glucose-6-phosphate isomerase	GPI
A0A0C4DFU2	Superoxide dismutase [Mn], mitochondrial	SOD2
A2RU48	Uncharacterized protein C12orf69	C12orf69
P17936	Insulin-like growth factor-binding protein 3	IGFBP3
Q01844	RNA-binding protein EWS	EWSR1
P49354	Protein farnesyltransferase/geranylgeranyltransferase type-1 subunit alpha	FNTA
B4E1Z4	Complement factor B	CFB
P99999	Cytochrome c	CYCS
D6RF35	Vitamin D-binding protein	GC
P00995	Pancreatic secretory trypsin inhibitor	SPINK1
Q13510	Acid ceramidase	ASAH1
Q9HBL0	Tensin-1	TNS1
Q99627	COP9 signalosome complex subunit 8	COPS8
Q9H0U4	Ras-related protein Rab-1B	RAB1B;RAB1C
E9PR30	40 S ribosomal protein S30	FAU
Q15436	Protein transport protein Sec 23 A	SEC 23 A
F8VW96	Cysteine and glycine-rich protein 2	CSRP2
J3QQ67	60 S ribosomal protein L18	RPL18
P16070	CD44 antigen	CD44
P15170	Eukaryotic peptide chain release factor GTP-binding subunit ERF3A	GSPT1
O15305	Phosphomannomutase 2	PMM2
P14415	Sodium/potassium-transporting ATPase subunit beta-2	ATP1B2
P22794	Protein EVI2A	EVI2A
Q9UBE0	SUMO-activating enzyme subunit 1	SAE1
O15143	Actin-related protein 2/3 complex subunit 1B	ARPC1B
O15144	Actin-related protein 2/3 complex subunit 2	ARPC2
O60306	Intron-binding protein aquarius	AQR
O60565	Gremlin-1	GREM1
O76038	Secretagogin	SCGN
O95497	Pantetheinase	VNN1
P01009	Alpha-1-antitrypsin;Short peptide from AAT	SERPINA1
P01011	Alpha-1-antichymotrypsin;Alpha-1-antichymotrypsin His-Pro-less	SERPINA3
P01275	Glucagon	GCG
P02766	Transthyretin	TTR
P05060	Secretogranin-1	CHGB
P05783	Keratin, type I cytoskeletal 18	KRT18
P07339	Cathepsin D	CTSD
P07814	Bifunctional glutamate/proline–tRNA ligase	EPRS
P08670	Vimentin	VIM
P09525	Annexin A4	ANXA4
P09936	Ubiquitin carboxyl-terminal hydrolase isozyme L1	UCHL1
P10645	Chromogranin-A	CHGA
P10909	Clusterin	CLU
P11021	78 kDa glucose-regulated protein	HSPA5
P16870	Carboxypeptidase E	CPE
P18085	ADP-ribosylation factor 4	ARF4
P22626	Heterogeneous nuclear ribonucleoproteins A2/B1	HNRNPA2B1
P23396	40 S ribosomal protein S3	RPS3
P25325	3-mercaptopyruvate sulfurtransferase;Sulfurtransferase	MPST
P29279	Connective tissue growth factor	CTGF
P31946	14-3-3 protein beta/alpha;14-3-3 protein beta/alpha, N-terminally processed	YWHAB
P35442	Thrombospondin-2	THBS2
P35579	Myosin-9	MYH9
P35580	Myosin-10	MYH10
P43034	Platelet-activating factor acetylhydrolase IB subunit alpha	PAFAH1B1
P46821	Microtubule-associated protein 1B;MAP1 light chain LC1	MAP1B
P48307	Tissue factor pathway inhibitor 2	TFPI2
P49189	4-trimethylaminobutyraldehyde dehydrogenase	ALDH9A1
P60174	Triosephosphate isomerase	TPI1
P60228	Eukaryotic translation initiation factor 3 subunit E	EIF3E
P60981	Destrin	DSTN
P62195	26S protease regulatory subunit 8	PSMC5
P62805	Histone H4	HIST1H4A
P63167	Dynein light chain 1, cytoplasmic	DYNLL1
P68366	Tubulin alpha-4A chain	TUBA4A
X6R8F3	Neutrophil gelatinase-associated lipocalin	LCN2
Q02790	Peptidyl-prolyl cis-trans isomerase FKBP4	FKBP4
Q06141	Regenerating islet-derived protein 3-alpha	REG3A
Q08380	Galectin-3-binding protein	LGALS3BP
Q12906	Interleukin enhancer-binding factor 3	ILF3
Q13162	Peroxiredoxin-4	PRDX4
A0A0B4J269	Tubulin beta-3 chain	TUBB3
Q14315	Filamin-C	FLNC
Q15008	26S proteasome non-ATPase regulatory subunit 6	PSMD6
Q15084	Protein disulfide-isomerase A6	PDIA6
Q15746	Myosin light chain kinase, smooth muscle	MYLK
Q15942	Zyxin	ZYX
Q6UX06	Olfactomedin-4	OLFM4
Q6VY07	Phosphofurin acidic cluster sorting protein 1	PACS1
Q8WU90	Zinc finger CCCH domain-containing protein 15	ZC3H15
Q8WUF5	RelA-associated inhibitor	PPP1R13L
Q96FJ2	Dynein light chain 2, cytoplasmic	DYNLL2
Q9BQE3	Tubulin alpha-1C chain	TUBA1C
Q9BRA2	Thioredoxin domain-containing protein 17	TXNDC17
Q9C040	Tripartite motif-containing protein 2	TRIM2
Q9C0C2	182 kDa tankyrase-1-binding protein	TNKS1BP1
Q9H8Y8	Golgi reassembly-stacking protein 2	GORASP2
Q9UHL4	Dipeptidyl peptidase 2	DPP7
Q9UNZ2	NSFL1 cofactor p47	NSFL1C
Q9Y570	Protein phosphatase methylesterase 1	PPME1

The proteins of cells grown on gelatin, flat-ZrO_2_ and ns-ZrO_x_ were analyzed by Perseus software. Among the 1379 proteins common to all conditions, 97 are differentially expressed (Anova test FDR 0.05).

Specific data sets were compared in order to identify the proteins that were differentially expressed under specific conditions, namely ns-ZrO_x_ versus gelatin, ns-ZrO_x_ versus flat-ZrO_2_ and flat-ZrO_2_ vs gelatin. The results can be seen in the supplementary material (Tables S1, S2, S3, S4, S5 and S6) with the Vulcano plots (Fig. S4) which reports differential expressed proteins between datasets (Welch’s t-test p value 0.0167). For each data set, bioinformatics analyses were carried out on the proteins differentially expressed in order to understand the effect of nanostructure.

**Figure 2:**
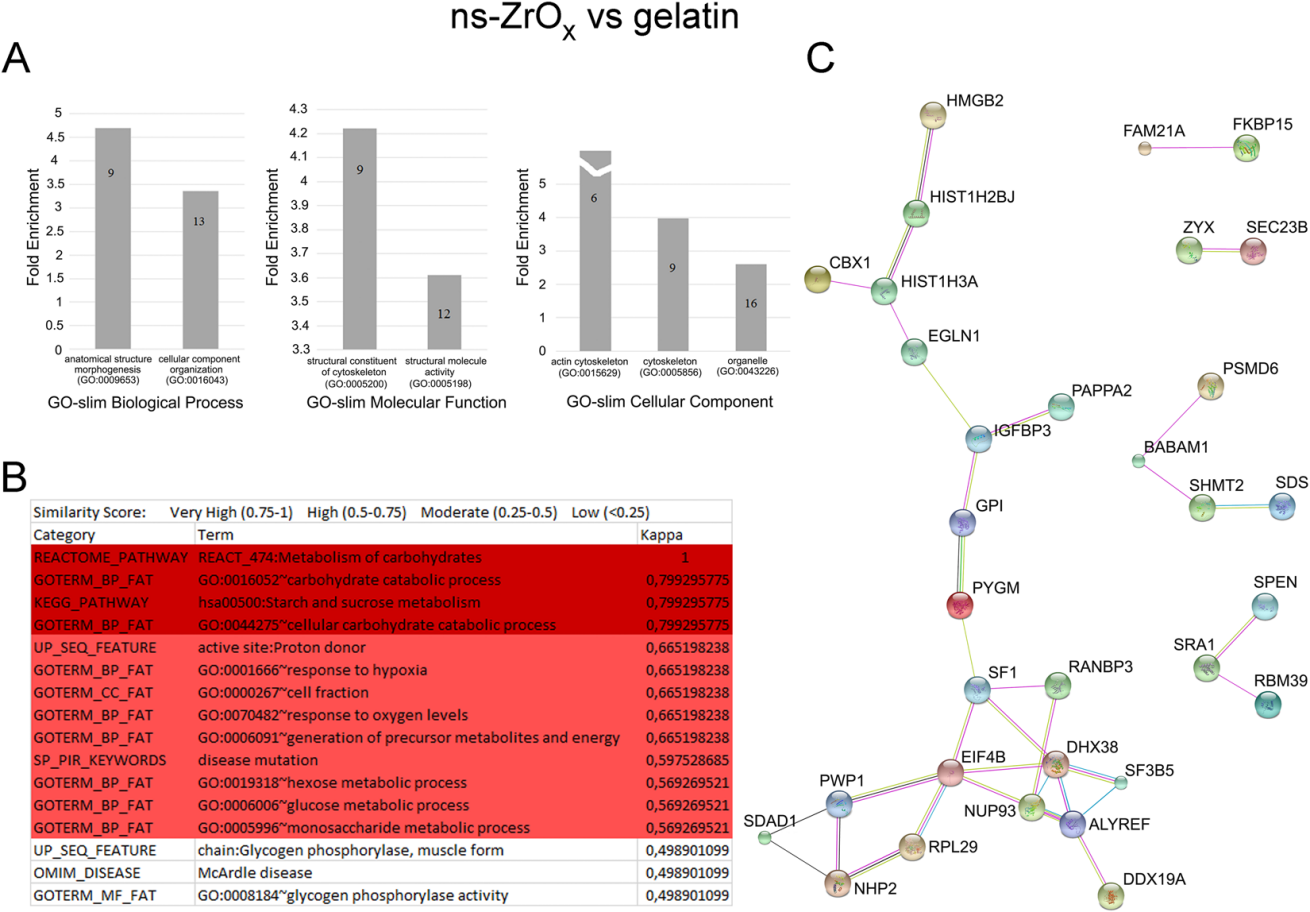
Bioinformatic analysis of proteins increased or exclusively expressed in cells grown on ns-ZrO_x_ in comparison to gelatin. (**A**) The proteins listed in Table S1 were classified in different biological processes according to the Gene Ontology classification system: GO-biological process (GOBP), GO-cellular component (GOCC) and GO-molecular function (GOMF) (GO) using Panther software. Functional grouping was based on p value ≤ 0.05. The numbers in the bars indicate the genes number for each category. (**B**) Reactome analysis. Only categories with confidence level Very High (K value 0.75–1) (Dark Purple) and High (K value 0.5–0.75) (Purple) are reported. (**C**) Network interactions of the same proteins data set were analyzed by String. Active interactions: text mining, experiments, databases; edges thickness indicates “confidence”. The proteins are indicated by the official gene symbol.

As regards the comparison between ns-ZrO_x_ and gelatin, the gene ontology (GO) analysis revealed that the ns-ZrO_x_ proteome was enriched for GO terms GO:0009653 anatomical structure morphogenesis and GO:0016043 cellular components organization elements, thus indicating a morphogenic process involving the actin cytoskeleton and intracellular organelles (Fig. 2A). The ns-ZrO_x_ substrate induced the upregulation of a number of chromatin associated proteins (GO:0031497), spliceosoma (GO:0005681) and heterogeneous nuclear ribonucleoprotein (GO:0030529) complexes, thus suggesting the activation of a robust program of transcriptional and translational regulation. Figure 2B,C show the bioinformatic analyses of the same data set in order to highlight protein-protein interactions and pathways. The reactome analysis (Fig. 2B) detected a highly significant enrichment in carbohydrate and glucose catabolic processes, response to hypoxia and oxygen level. In accordance, the interaction networks determined with the STRING analysis showed that, among the various categories of proteins modulated by ns-ZrO_x_ substrates there was an increase in the expression of proteins involved in the modulation of cell metabolism (EGLN1/PHD2, PYGM and GPI) as well as in ER-to-Golgi membrane trafficking (mediated by the SEC 23B protein). The IGF proteins (PAPPA2 and IGFBP3) and steroid receptor (SF1, SPEN, SRA-1 and RBM39) signaling pathways were also increased by ns-ZrO_x_ (Fig. 2C).

**Figure 3:**
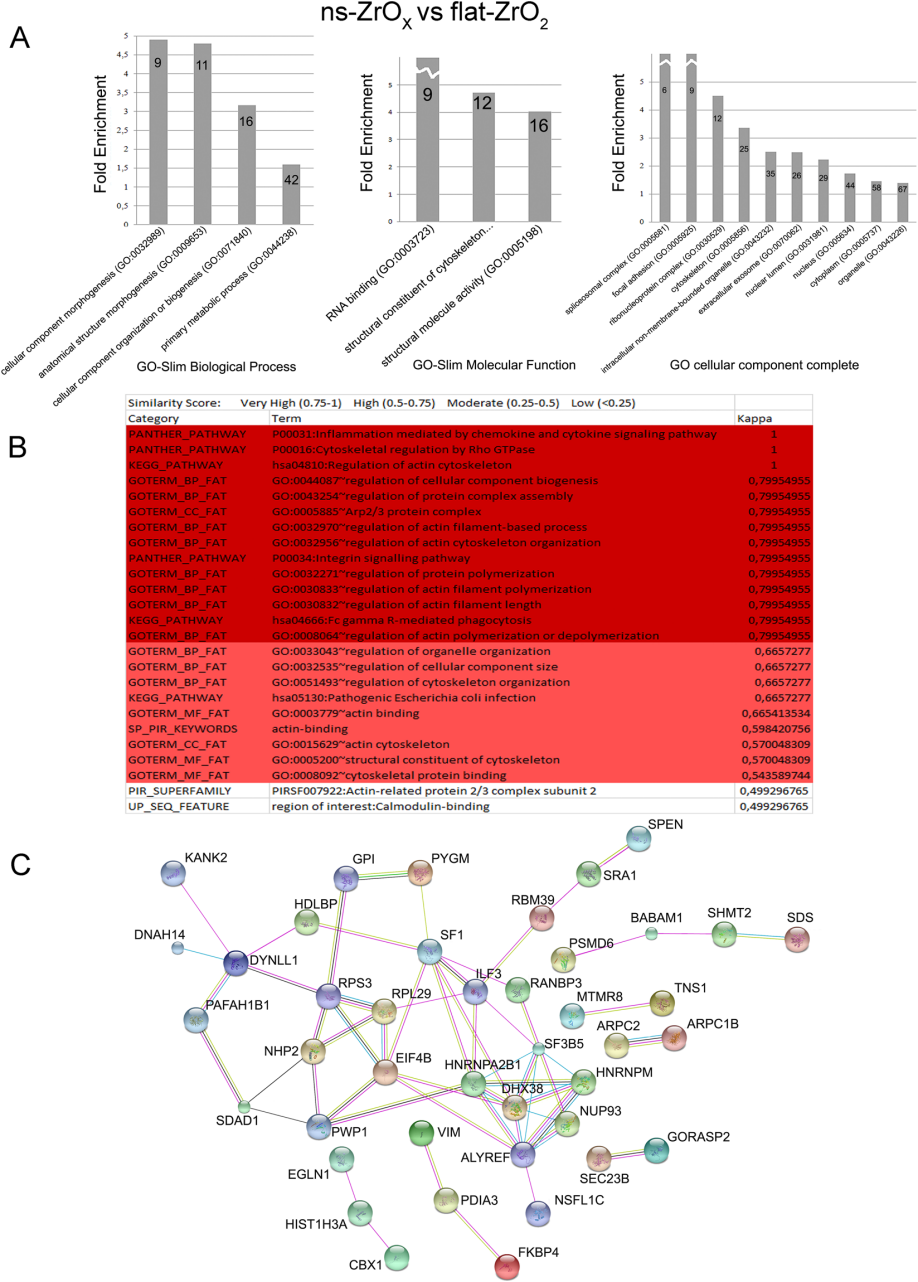
Bioinformatic analysis of the proteins increased or exclusively expressed in cells grown on ns-ZrO_x_ in comparison to flat-ZrO_2_. (**A**) The proteins listed in Table S3 were classified into different biological processes according to the Gene Ontology classification system GO-biological process (GOBP), GO-cellular component (GOCC) and GO-molecular function (GOMF) using Panther software. Functional grouping was based on p value ≤ 0.05. The numbers in the bars indicate the number of genes for each category. (**B**) Reactome analysis. Only categories with confidence level: Very High (K value 0.75–1) (Dark Purple) and High (K value 0.5–0.75) (Purple) are reported. (**C**) Network interactions in the same proteins data set were analyzed by String. Active interactions: text mining, experiments, databases; edges thickness indicates “confidence”. The proteins are indicated by the official gene symbol.

A GO analysis of the proteins only expressed and upregulated in ns-ZrO_x_ compared to flat-ZrO_2_ substrates revealed a very similar effect on the proteomic pattern to that observed when comparing ns-ZrO_x_ and gelatin, and the enrichment of the GO cellular component terms related to morphogenesis (GO:0032989), anatomical structure morphogenesis (GO:0009653), cellular component organization or biogenesis (GO:0071840) and metabolic processes (GO:0044238) (Fig. 3A). Protein-protein interaction analysis (Fig. 3B,C) revealed similar networks, thus emphasizing the fundamental role of nano-topography in the induction of the morphogenic program.

Interestingly, among the upregulated or uniquely expressed proteins on ns-ZrO_x_, we found focal adhesion molecular components (GO:0005925) and important proteins for actin polymerization (GO:0005856) such as TNS1, ARPC2, ARPC1B, DYNLL1, DNAH14, KANK2 protein complexes, as well as proteins involved in controlling nuclear architecture (GO:0031891) and nuclear import/export (GO:0005634), thus suggesting the activation of a mechanotransductive signaling pathway. Moreover, there was a statistically significant enrichment of the integrin signaling pathway (P00034) (Fig. 3B).

### Quantitative immunofluorescence analysis confirms the activation of a mechanotransduction pathway in islets grown on ns-ZrO_x_

**Figure 4:**
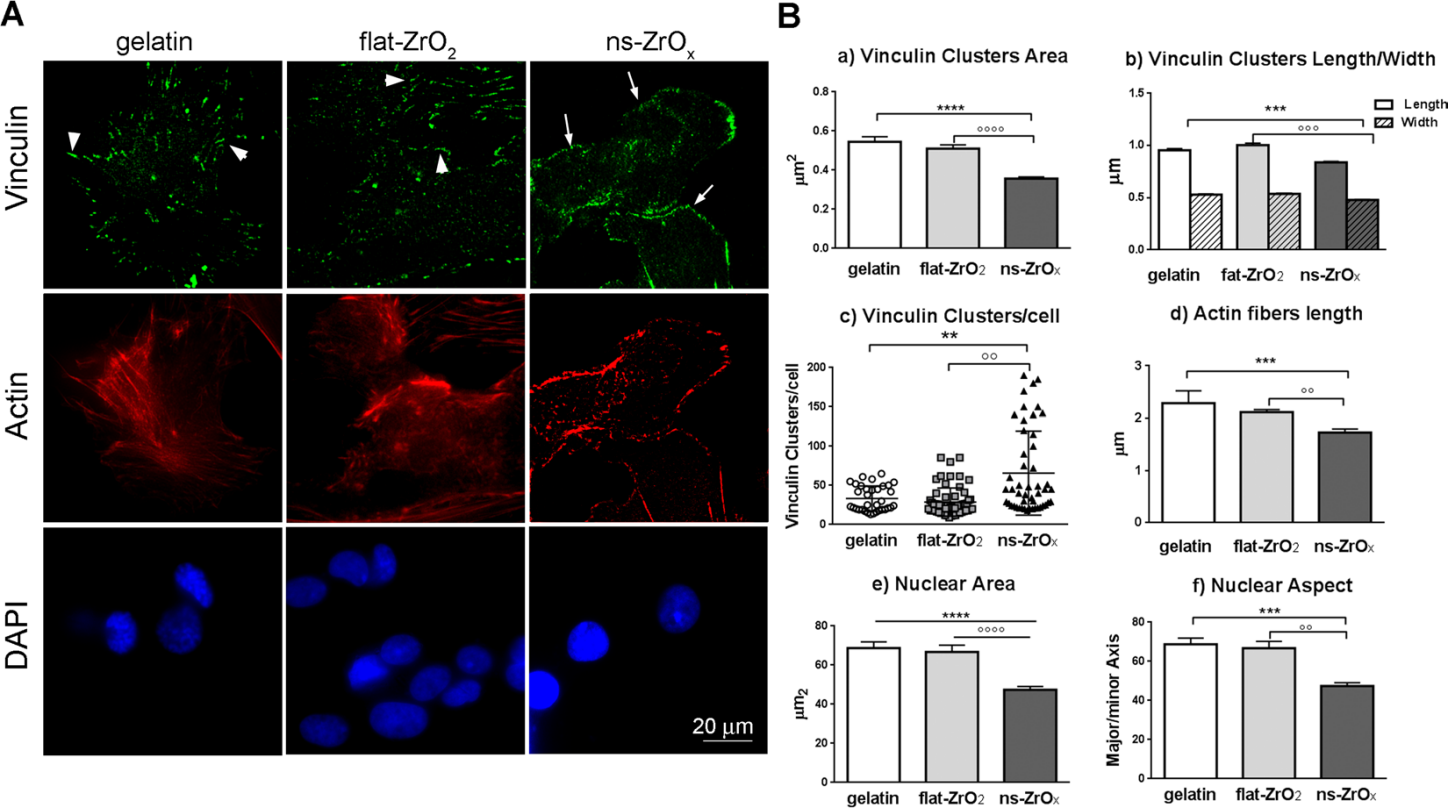
Nanostructured zirconia substrates promote the activation of a mechanotransduction pathway. (**A**) Cells, grown on different substrates for 15 days, were triple stained with anti-vinculin antibody (green), phalloidin (actin, red) and DAPI (blue). Representative epifluorescence (actin and DAPI) and TIRFM (vinculin) images are shown. Bar: 20 μm. Arrows indicate focal complexes, arrowheads indicate focal adhesion. (**B**) Quantitative analyses of adhesive complexes, actin fibers organization and nuclear architecture of cells grown on different substrates. (a,b) Vinculin-positive clusters area, length and width; (c) number of vinculin clusters per cell; (d) cytoskeletal actin fibers length; (e,f) nuclear area and aspect (major/minor axis). Bars illustrate the average responses ± SE (N = 40–100 cells for each substrate) in two different islet preparations. (***p < 0.005, ns-ZrO_x_ *vs* gelatin; °°p < 0.01, °°°p < 0.005, ns-ZrO_x_ *vs* flat-ZrO_2_).

In order to demonstrate the activation of a mechanotransduction pathway in β-cells, the organization of cell-substrate adhesive contacts and actin cytoskeleton were analyzed (Fig. 4A). TIRF microscopy showed discrete vinculin-positive clusters distributed at the cell periphery in cells grown on gelatin and flat-ZrO_2_. On ns-Zr_x_ substrates the organization and the distribution of adhesive contacts differed significantly. Diffuse punctate staining, indicating nanoclusters or small focal contacts, was visible in the cell, but organized focal adhesions like those observed on flat substrates did not form. The observations were confirmed by the quantitative analyses (Fig. 4Ba–c). There were fewer vinculin clusters per cell on gelatin and flat-ZrO_2_ cells yet they had significantly larger areas and dimensions than those observed on ns-ZrO_x_.

A similar trend was observed for the correlated formation of high order actin filament structures (Fig. 4A,Bd). Stress fibers were mainly detected on gelatin and flat-ZrO_2_, but seldom formed on ns-ZrO_x_, where actin was organized in bundles at the cell-cell contacts. Cell-cluster formation was mainly detected on nanostructured substrates probably due to this actin reorganization.

It has been suggested that mechanical forces acting via integrin adhesions and the actin cytoskeleton can induce the modification of the nuclear envelope, chromatin architecture and hence the transcriptional program^37^. This prompted us to investigate the nuclear architecture. Quantitative analyses of DAPI stainings revealed the presence of smaller nuclear structures with increased roundness (major/minor axis) on ns-ZrO_x_ compared to gelatin and flat-ZrO_2_ (Fig. 4Be,f).

Taken together, our data suggest that the morphogenetic program evoked by the nanostructure is fueled by the activation of a mechanotransductive pathway involving adhesive contacts, the actin cytoskeleton and modification of the nuclear architecture.

### Cluster-assembled zirconia substrates prevent β-cell death by apoptosis and necrosis and modulate NF-κB and HIF1α signaling pathways

Bioinformatic analysis revealed the expression of anti-aging and anti-apoptotic proteins such as BABAM1, PSMD6 and SHMT2 and the upregulation of known proteins involved in pro-survival and pro-differentiation signaling pathways, such as the Insulin Growth Factor (IGF) and the steroidogenic signal pathways.

**Figure 5:**
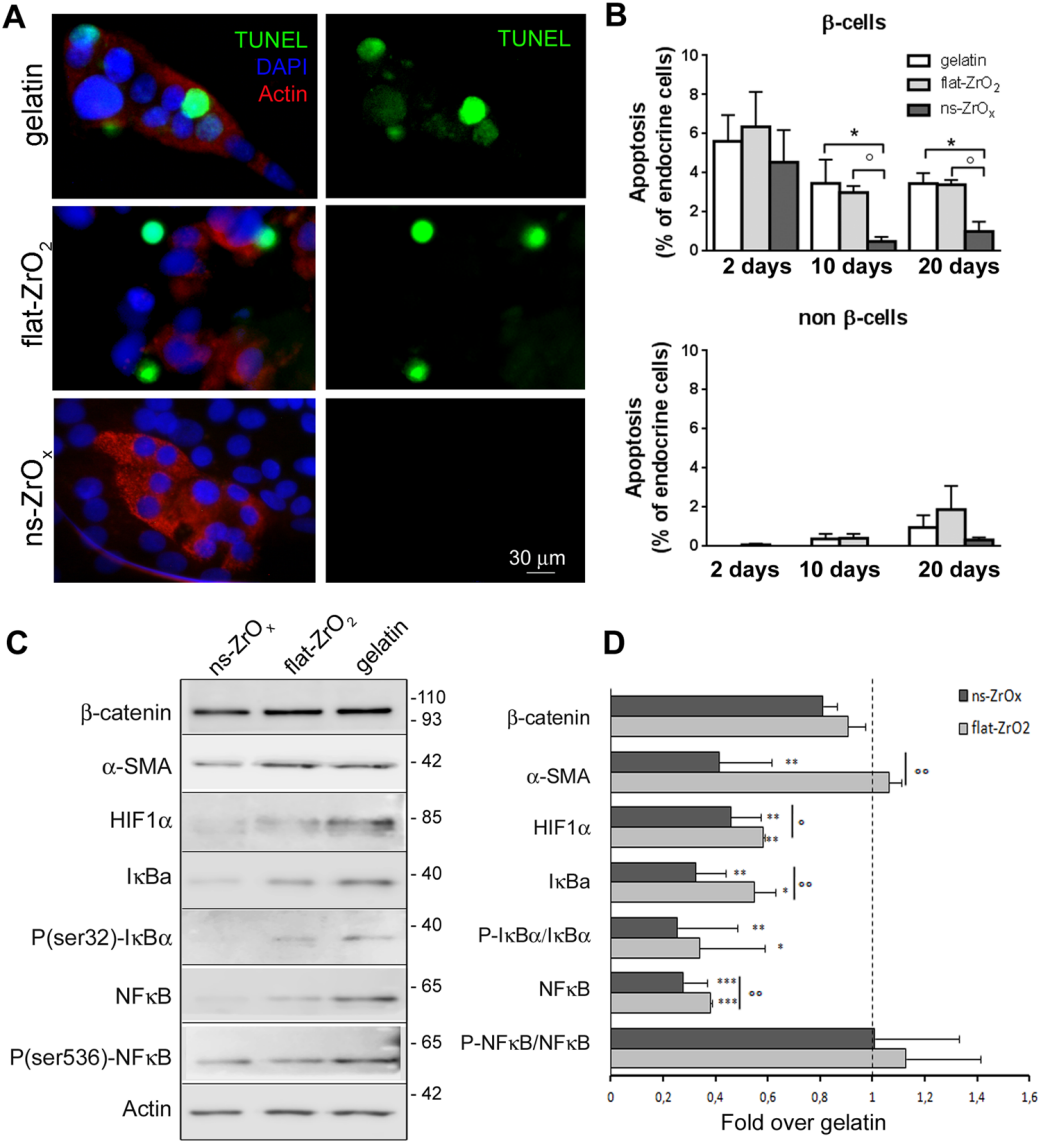
Nanostructured Zirconia substrates prevent β-cells death through modulation of hypoxia and NF-κB pathways. (**A**) Representative immunofluorescence images of islets triple stained with TUNEL (green), anti-insulin antibody (red) and DAPI (blue). Bar = 30 μm. (**B**) Quantification of β-cell apoptosis by TUNEL assay in human islets grown on different substrates for 2, 10 and 20 days. β-cell apoptosis represents the percentage of TUNEL- and insulin-double positive cells over total insulin-positive cells; non β-cell apoptosis represents the percentage of TUNEL-positive cells over total DAPI-positive and insulin-negative cells. The experiment was performed in triplicate, with three different islet preparations. A minimum of 100 cells for islet preparation was counted (*p < 0.05 *vs* gelatin; °p < 0.05 *vs* flat-ZrO_2_). (**C**) Western-blotting analysis of hypoxia and NF-κB pathways selected proteins in islets grown on the indicated substrates for 20 days (15 μg protein/sample). On the right, the proteins molecular weight in kDa is reported. (**D**) Quantitative analysis of protein expression shows that flat-ZrO_2_ and ns-ZrO_x_ substrates downregulate the hypoxia and NF-κB pathways. Data (mean values ± S.E.; n = 5 independent experiments) are expressed as fold change over gelatin (dashed line). (*p < 0.05, **p < 0.01, ***p < 0.005, ns-ZrO_x_ *vs* gelatin. °p < 0.05, °°p < 0.01, ns-ZrO_x_ *vs* flat-ZrO_2_).

In order to confirm the activation of pro-survival and anti-apoptotic pathways, we evaluated the rate of β-cell apoptosis by TUNEL assay in islets grown on different substrates for 2, 10 and 20 days (Fig. 5A). After two days in culture, β-cell apoptosis was identical and relatively high for all three substrates (7.3 ± 0.5%), probably due to the isolation procedure and the trypsinization process. However, over time in culture, β-cell apoptosis was three fold lower in islets grown on ns-ZrO_x_ than on the other supports (1.5 ± 0.2%; p < 0.05) (Fig. 5B).

Besides apoptosis, necrosis was also significantly reduced on ns-ZrO_2_ relative to flat-ZrO_2_ or gelatin (two fold decrease; p < 0.005) (Suppl. Fig. 5), thus indicating that ns-ZrO_x_ substrates prevent β-cell death.

Hypoxia and activation of pro-inflammatory pathways are the key determinants of β-cell death in culture systems^38^. Interestingly, ns-ZrO_x_ increases the expression of the oxygen sensor EGLN1/PHD2, a prolyl hydroxylase domain protein which catalyzes the hydroxylation of proline residues in an oxygen-dependent manner and target proteins for degradation through the proteasomal complex^39^. Among the PHD2 target proteins are hypoxia inducible factor 1α (HIF-1α) and IκB kinase 2 (IKK2), the major regulator of NF-κB activation. Western blot experiments (Fig. 5C,D) performed with cells grown on different substrates confirmed the proteome analysis and revealed decreased expression of HIF1α, IκBα and NF-κB and decreased phosphorylation of IκBα in cells grown on zirconia substrates compared to gelatin. These modifications were significantly more prominent in islets grown on ns-ZrO_x_ substrates than on flat-ZrO_2_.

In line with these findings, the proteome analysis showed a decreased expression of the proteins involved in the activation and regulation of NF-κB (TICAM1 (Tables S2 and S6), USP51 (Tables S4 and S5) and COPS8 (Table S4)) and upregulation of proteins promoting NF-κB degradation such as the ribosomal protein S3 (rpS3) and the N-ethylmaleimide-sensitive factor L1 cofactor (NSFL1C)^40^. Proteins with anti-inflammatory properties, such as the Interleukin enhancer-binding factor 3 (ILF3) which controls interleukin 2 transcription are also upregulated by ns-ZrO_x_.

In order to verify if ns-ZrO_x_ substrates promote β-cell proliferation, islet cultures on different substrates were stained with Ki67 at 5 and 10 days in culture (Suppl. Fig. S6A and B). At both time points we found an higher percentage of proliferating β-cells on ns-ZrO_x_ than on controls substrates, although the difference was not statistically significant. As expected, the rate of β-cell proliferation was two-fold lower than that of non-endocrine cells under all experimental conditions (p < 0.05). A similar trend was observed when carrying out a FACS analysis on Ki67-PDX1 double-positive cells grown for 5 and 10 days on different substrates (Suppl. Fig. S6C–E).

Taken together, these data indicate that cluster-assembled zirconia substrates support β-cell proliferation and preserve β-cell survival and differentiation, by reducing hypoxia and islet inflammation.

### Cluster-assembled zirconia substrates preserve insulin granules and the glucose-stimulated insulin release

**Figure 6:**
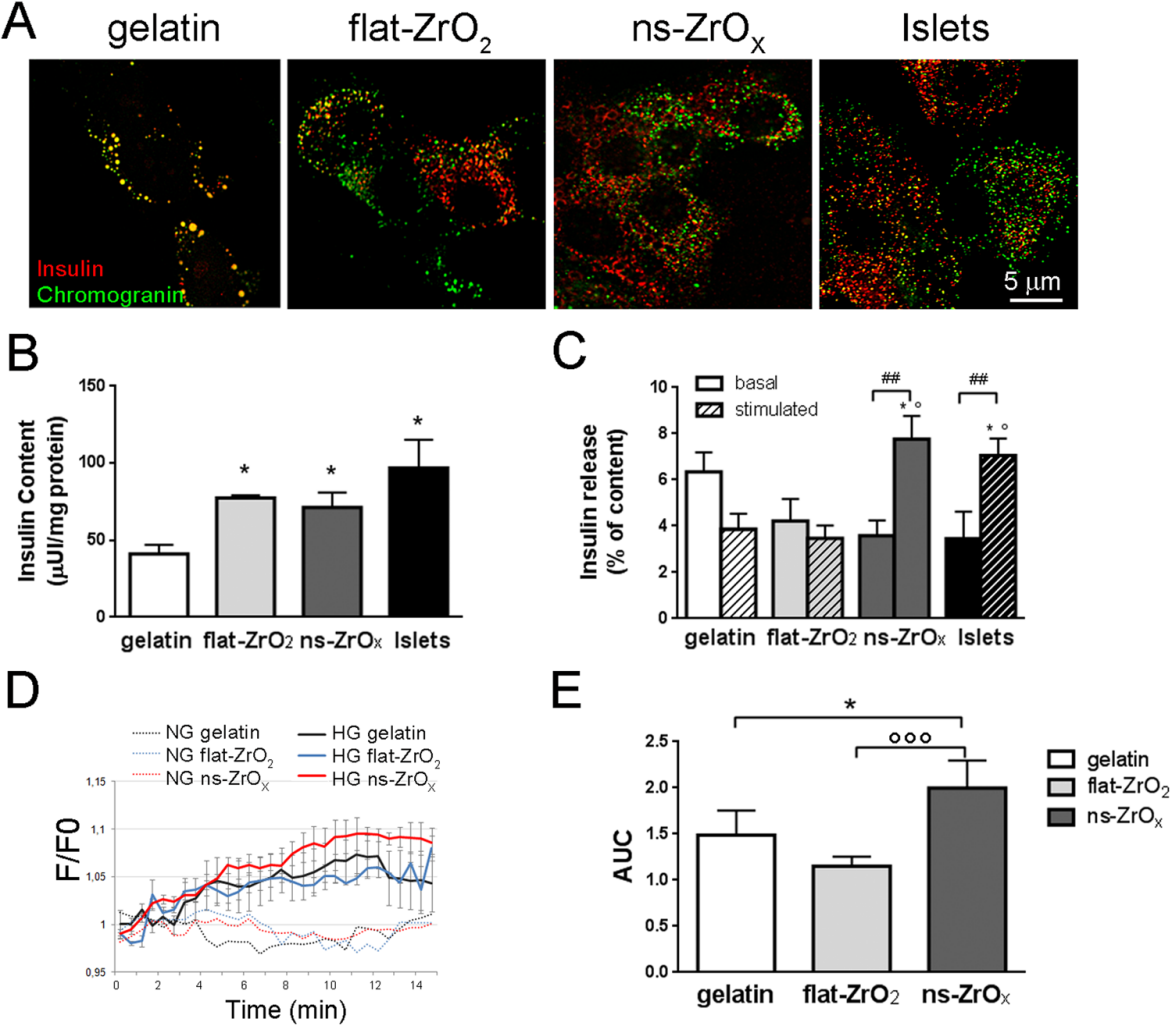
Nanostructured substrates preserve β-cell function in long term cultures. (**A**) Insulin granules density and distribution were evaluated by double immunofluorescence stainings with anti-insulin (red) and anti-chromogranin A (green) antibodies. The yellow-orange staining indicates colocalization between the two markers. Representative confocal images are shown. Bar = 5 μm. (**B**) The insulin content was evaluated in human islets cultured for 20 days on the indicated substrates or in freshly-isolated intact islets (three days old islets) (islets, black bars). Data (mean ± SD) are expressed as μUi of insulin per mg of protein (n = 3, in duplicate) (*p < 0.05; *vs* gelatin). (**C**) The insulin secretory response in basal (3.3 mM glucose) and stimulated (16.7 mM glucose) conditions was evaluated in human islets cultured for 20 days on the indicated substrates or in freshly isolated intact islets. Data (mean ± SD) are expressed as % of insulin content (n = 3, in duplicate). (*p < 0.05, *vs* gelatin; °p < 0.05, *vs* flat-ZrO_2_; ##p < 0.001 stimulated *vs* relative basal). (**D**) The cells were loaded with the Ca^2+^ indicator Fluo3 and Calcium imaging was performed under basal (NG, 3.3 mM glucose) (dotted lines) and stimulated (HG, 20 mM glucose) (continue lines) conditions. The time course of changes in fluorescence signals (F/F0) induced by glucose application (bars over traces) were recorded from islets grown on gelatin (black), flat-ZrO_2_ (blue) and ns-ZrO_x_ (red). The curves illustrate average responses ± S.E. from four different islet isolations (N = 20 cells for each experiment) (p < 0.005 ns-ZrO_x_ *vs* gelatin; P < 0.0001 ns-ZrO_x_ *vs* flat-ZrO_2_). (**E**) Area Under the Curves (AUC) of experiments reported in D (*p < 0.05 ns-ZrO_x_ *vs* gelatin; °°°p < 0.001 ns-ZrO_x_ *vs* flat-ZrO_2_).

The proteome of cells grown on zirconia cluster-assembled films revealed an upregulation of proteins involved in the regulation of membrane trafficking, typically observed in fully differentiated β-cells (i.e. GORASP2, SEC 23B). In order to determine the maturation of β-cells in culture, we evaluated the distribution of insulin secretory granules in cells grown in culture for 20 days. β-cells grown on ns-ZrO_x_ contained several insulin-positive granules, distributed in the cytosol and in proximity of the cell membrane (Fig. 6A). A similar distribution was detected on flat-ZrO_2_ and in intact islets (three day-old intact islets). Conversely, in islets grown on gelatin, β-cells contained few insulin-positive granules of increased dimensions in the perinuclear region, thus indicating that zirconia substrates preserve the mature β-cell phenotype and prevent β-cell dedifferentiation.

In order to further support a role of cluster-assembled substrates in preserving β-cell function, the insulin content and the insulin secretion under basal (3.3 mM glucose) and stimulated (16.7 mM glucose) conditions were measured by means of an ELISA assay (Fig. 6B,C). The insulin content (Fig. 6B) was significantly higher in islets grown on zirconia substrates (both flat and nanostructured) compared to gelatin-grown cells, thus supporting increased β-cell survival and maturation on zirconia substrates. Islets grown on ns-ZrO_x_ presented a lower basal insulin release and an intact GSIS response, comparable to that observed in freshly-isolated intact islets. Conversely in islets grown on flat-ZrO_2_ or gelatin the insulin secretion under basal conditions was similar or paradoxically higher than that observed under high glucose stimulation (Fig. 6C). These secretory defects (increased basal insulin secretion, reduced stimulated insulin release) were observed in “dedifferentiated” and “stressed” human islets after chronic exposure to high glucose concentrations^41^. The insulin granule assembly and exocytosis are a Ca^2+^-dependent mechanism. We monitored changes in intracellular Ca^2+^ by Ca^2+^-imaging with Fluo3 and we found that islets grown on ns-ZrO_x_ showed increased integrated Ca^2+^ currents after glucose stimulation than the cells grown on gelatin and flat-ZrO_2_ (45% and 70% increase, respectively) (Fig. 6D,E). Taken together, these data indicate that the ns-ZrO_x_ not only supports β-cell survival but also promotes β-cell differentiation and preserves the glucose stimulated insulin secretion.

## Discussion

Our data show that ns-ZrO_x_ substrates support long-term culture of human islets *in vitro* by increasing β-cells survival and preserving β-cell differentiation (Figs 1, 5 and 6).

The increased β-cell survival on cluster-assembled substrates was due to decreased apoptosis and necrosis. Furthermore, only the islet β-cells grown on nanostructured surfaces maintained an intact secretory apparatus, with several dispersed insulin granules and retained the glucose-sensitive insulin secretion, thus indicating that these culture conditions also preserve β-cell differentiation.

A proteomic approach was used to identify the molecular mechanisms modulating islet cell behaviour. We looked for proteins that were differentially expressed in human islets cultured for 20 days on ns-ZrO_x_ as compared to islets from the same donor, cultured under identical conditions on gelatine. We then compared the protein profile of islets grown on ns-ZrO_x_ with those of flat-ZrO_2_ in order to evaluate the contribution of topography. One of main issues of islet proteome profiling is the potential contamination of exocrine acinar tissue in islet preparations. The islet preparation selected for proteomic analysis contained minor exocrine contamination (80–90% islet preparation purity). Furthermore, after comparing the data with the islet high quality proteome reported in^42,43^, the proteins deemed to be possible contaminants were filtered out, thus providing confidence to the approach.

Among the uniquely expressed or upregulated proteins in ns-ZrO_x_ cultured islets (compared to gelatin) there were proteins controlling transcription and translation, thus indicating a significant increase in protein synthesis rates. This effect was due to the nanostructured topography because a similar trend of proteins was found to be expressed or over-expressed in cells cultures grown on ns-ZrO_x_ and not in those grown on flat-ZrO_2_.

Our data clearly indicate that the nanostructure of cluster-assembled films supports the up-regulation of cytoskeleton associated proteins such as KANK proteins (Tables S1 and S3) and TENSIN, the actin-crosslinking protein (TNS1) (Table S3) at focal adhesions^44^. Molecular motor proteins such as dynein light chains DYNLL (Table S3), axonemal dynein (DNAH14) (Tables S1, S3 and S5), which amplify the mechanical force generated by primary cilia in sensory neurons^45^ and the dynein-regulating protein PAFAH1B1 (LIS1) were also detected. Interestingly, these proteins are part of a recently-described mechanotransductive pathway operating in several cells to direct their adaptation to changes in extracellular cues^44^. Moreover, new protein expressions and up-regulations controlling the nuclear envelope architecture and the nuclear import/export were found (i.e. NUP93, RANBP3) (Tables S1 and S3).

**Figure 7:**
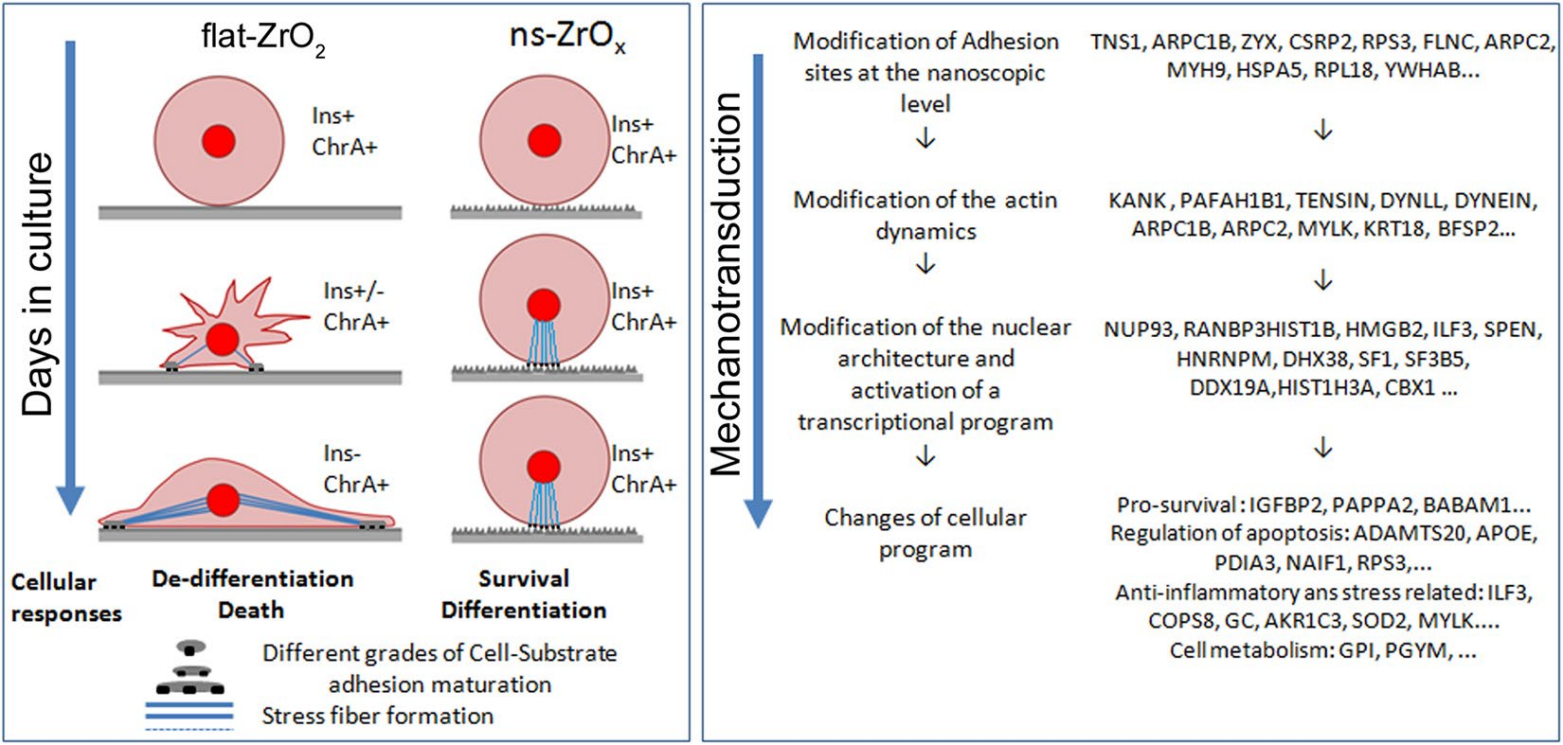
β-cells relay on nanotopography features to regulate their differentiation and survival. Modification of β-cell shape and insulin expression in cells grown on flat-ZrO_2_ or ns-ZrO_x_ during the *in vitro* culture. Ins: insulin, ChrA: chromogranin A, −/+ insulin or chromogranin A expression. Only β-cells grown on ns-ZrO_x_ maintain their circularity and activate a survival and pro-differentiation program. The process is supported by a mechanotransduction pathway, driven by the nanostructured topology, via remodeling of the actin cytoskeleton, nuclear architecture and activation of a transcriptional program that promotes β-cell survival and differentiation. Examples of genes involved in the different processes are reported (the complete list of genes is reported in Supplementary Tables).

These data highlight the presence of a mechanotransductive pathway in pancreatic islets that senses the extracellular environment and possibly by modifying the actin cytoskeleton, changes the tension on the nuclear envelope which in turn modulates the gene transcription and cellular remodelling program^37,44^ (Figs 4 and 7). As a result we found new expressions of critical proteins involved in the organization and the control of the secretory pathway (i.e. SEC 23B, GORASP2) (Tables S1 and S3), in the promotion of β-cell survival and in the modulation of cell metabolism, thus supporting our experimental data on increased β-cell viability and function.

Among the various categories of proteins linked to ns-ZrO_x_ substrates we found increased expression of proteins with anti-aging and anti-inflammatory properties and proteins involved in the activation of pro-survival signalling pathways such as IGF and steroid signalling pathways. Both signaling pathways are known to promote β-cell survival and function and alteration of their components may contribute to the development of type 2 diabetes^46–49^.

Our experimental data not only indicate decreased apoptosis but also highlight the presence of a fully differentiated β-cell phenotype, with an intact biosynthetic route for insulin packaging and a signalling machinery capable of coupling glucose-sensing to insulin secretion.

The increased organization of the early secretory apparatus observed on ns-ZrO_x_ may be due to the expression of a number of proteins involved in membrane trafficking, among which TGN38B and GORASP2 that are required for the ER to Golgi trafficking of insulin. TGN38B/SEC 23B is a GTPase-activating protein abundantly expressed in professional secretory tissues; its deficiency in embryos causes the accumulation of proteins in the endoplasmic reticulum and activation of the pro-apoptotic pathway of the unfolded protein response, leading to defects in pancreas development^50^. The Golgi reassembly stacking protein 2 GORASP2/GRASP55 is a matrix protein involved in Golgi stack and ribbon formation^51^ and interference with its expression strongly inhibits intra-Golgi transport of large cargoes like insulin.

The preserved glucose stimulated insulin secretion (GSIS) linked to ns-ZrO_x_ may be due to the expression of the voltage-dependent calcium channel gamma-like subunit (TMEM37) (Tables S1 and S3), an auxiliary subunit of calcium channel complex which modifies the time course of current activation and inactivation of the P/Q type alpha (1A) subunit, thus directly controlling insulin release^52^. Another interesting protein is the prolyl hydroxylase domain protein EGLN1 also known as PHD2 (Tables S1 and S3), expressed in the pancreas^53^. EGLN1 catalyzes the hydroxylation of proline residues in an oxygen-dependent manner and targets proteins for degradation. One of the PHD substrates is α-keto glutarate (αKG) which has been proposed to act as insulin secretagogue in the K_ATP_ channel‐independent or the amplifying GSIS pathway. Interestingly, inhibition of αKG‐dependent hydroxylases blocks GSIS^53,54^, thus emphasizing the important role this enzyme plays in controlling insulin secretion.

We favour the idea that the nanostructure may improve β-cell survival and differentiation through a mechanotransduction process coupled with a fine modulation of the redox-state of the cells. Indeed, a number of proteins functioning as red-ox and oxygen sensors correlate with ns-ZrO_x_, including the above-mentioned EGLN1/PHD2 protein which targets HIF-1α^39,55^. In normoxia, HIF-1α subunit is marked for proteasomal degradation through hydroxylation by EGLN1/PHD2 and its interacting partner HIF-1β can drive the expression of genes involved in β-cell differentiation and function^55^. Under hypoxic conditions, the stabilization of HIF-1α leads to the formation of a heterodimer complex with HIF-1β and diverges the latter from the control of β-cell differentiation program^55,56^. In islets grown on ns-ZrO_x_ there is an upregulation of the oxygen sensor EGLN1/PHD2 which may lead to HIF-1α downregulation, increased metabolic activity and induction of the differentiated endocrine phenotype, in perfect agreement with our results. In line with this as well as the downregulation of HIF-1α, we also detected a decreased expression of alcohol dehydrogenase (CFB) (Table S2) which is normally abundantly expressed under hypoxic conditions and the upregulation of L-serine dehydratase (SDS) (Tables S1 and S3) which is the enzyme that catalyzes the conversion of L-serine into ammonia and pyruvate, whose gene expression is oxygen dependent^57^.

Interestingly, while PHDs regulate HIF-1α protein stability, EGLN1/PHD2 is subject to feedback up-regulation by HIF-1α, given that the EGLN1/PHD2 gene contains a hypoxia-response element (HRE) that can be recognized by HIF-1α^58^. This feedback mechanism may be important to ensure a basal level of HIF-1α expression required for activating a metabolic response to oxidative stress during culture conditions. In line with this possibility, there is also upregulation of proteins usually induced by HIF-1α to reduce ROS production, modulate oxygen consumption and prevent possible hypoxic injuries.

Interestingly, the nanostructure induces a significant enrichment of GO terms and interactions related to the catabolic metabolism of carbohydrates increasing the expression of proteins involved in the adaptive metabolic mechanisms linked to oxygen bioavailability: glucose-6-phosphate isomerase (GPI), lactase-phlorizin hydrolase (LCT), commonly known as Lactase and mio-glycogen phosphorylase (PYGM).

Moreover, one of the proteins upregulated by the nanostructure is mitochondrial serine hydroxymethyltransferase (SHMT2) (Tables S1 and S3). Its activity limits pyruvate kinase activity and reduces oxygen consumption, thus creating a metabolic state that confers a significant survival advantage to cells in poorly oxygenized regions^59^. By transferring a methyl group from serine to tetrahydrofolate (THF), the protein can initiate the degradation of serine to CO_2_ and NH_4_^+^, producing glycine and methylene-THF which is essential for producing NADPH, thus increasing the NADPH/NADP ratio required to replenish GSH and repress ROS generation in mitochondria.

Alternatively, EGLN1 may also act through NF‐κB, given that the IκBα kinase can be directly hydroxylated by the enzyme^60^. With an NF‐κB-mediated mechanism, in the brain PHD1, an EGLN1 isoform, fine tunes biochemical pathways and energy metabolism, according to oxygen availability^61^. In β-cells NF‐κB controls inflammation and cytokine-mediated β-cell apoptosis, which are the key components of β-cell dysfunction in diabetes^38^. In line with this possibility, we found decreased phosphorylation and expression of NF‐κB and upregulation of anti-inflammatory pathways.

## Conclusions

In summary, our results show that ns-ZrO_x_ substrates favour long-term culture of human islets by supporting β-cell survival and preserving β-cell differentiation and function. This phenomenon is closely related to nanoscale surface topography, since only cells grown on cluster-assembled films maintain the fully differentiated and functional phenotype.

We suggest that by activating a mechanotransduction pathway coupled to modulation of the redox state of the cell, the ns-ZrO_x_ fine tunes the expression and function of EGLN1/PHD, which in turn orchestrates, via HIF-1α and NF‐κB pathways, an adaptive glucose metabolism response which preserves β-cell differentiation and function under culture conditions.

By exploiting the unique properties of cluster-assembled films, we can gain a better understanding of the interaction between cells and nanostructures and investigate how mechanical forces contribute to β-cell fate and function. This will enable us to define novel molecular targets of intervention for preventing β-cell failure in diabetes mellitus and will provide valuable insight for developing substrates with well-defined nanotopography that can improve the efficacy of islet cell transplantation therapies.

## Materials and Methods

### Nanostructured Substrates Fabrication

Cluster-assembled films were grown on glass cover slides by depositing a seeded supersonic beam of ZrO_x_ clusters produced by a pulsed microplasma cluster source (PMCS) under high vacuum conditions. A detailed description of the deposition technique is provided in ref.^30^.

In short, the supersonic cluster beam deposition (SCBD) basically consists in: (i) sputtering of a zirconium rod placed in the PMCS by aerodynamically-confined argon plasma ignited by an high-voltage electrical discharge; (ii) condensation of the ablated atoms in clusters; (iii) expansion of the argon-cluster mixture through a nozzle into a vacuum in order to form a seeded supersonic beam and collection of the cluster beam on a substrate mounted on a manipulator placed perpendicularly to the beam trajectory. The film obtained is partially oxidized due to the presence of residual oxygen deposition apparatus; further oxidation takes place upon exposure to air, resulting in a substoichiometric ZrO_x_ (x < 2) film^35^.

Thanks to the correlation between the thickness and the roughness of the deposit, it is possible to obtain films with controlled nanoscale roughness as a function of the film thickness^31^. Some samples, indicated as flat substrates, of fully oxidized zirconia films with very low rms roughness (<0.5 nm) were produced by electron beam evaporation of ZrO_2_ targets as controls.

### Surface Morphology Characterization of Substrates by AFM

The surface morphology of the nanostructured films was characterized by Atomic Force Microscopy (Multimode Nanoscope IV). The measurements were taken in tapping mode (single-crystal silicon tips having nominal radii of curvature in the range 5–10 nm and cantilever resonance frequency in the range of 200–300 kHz). 2 μm x 1 μm images were acquired with scan rates of 1.5–2 Hz and sampling resolution of 2048 × 512 points.

Cell culture experiments were performed on substrates with 15 and 20 nm of rms roughness. Further details on AFM characterization of cluster assembled nanostructured films can be found in ref.^35^.

### Human Islet Isolation and culture on nanostructured substrates

The islets used in this study were isolated in Milan (Niguarda Ca’ Granda) from cadaveric multiorgan donors with no medical history of diabetes or metabolic disorders according to the procedure described by Ricordi *et al*.^62^.

Within three days from isolation, 600 pancreatic islets were dispersed into small cell clusters and single cells by brief digestion (22 °C for 3 min) in phosphate saline buffer (PBS) supplemented with 1 mg/mL trypsin and 0.25 mg/mL DNAse. Trypsin digestion was halted by adding PBS to a total volume of 2 mL. The islets were collected by centrifugation (5 min at 350 × g) and resuspended in 600 µL of complete medium. 100 µL aliquots of islet suspension were then transferred onto glass coverslips (30 islets/mm^2^) coated with gelatin (50 mg/mL), ns-ZrO_x_ (15 and 20 nm rms) and flat-ZrO_2_. After 6 hours, complete medium (RPMI culture medium containing 5.5 mmol/L glucose, 10% heat-inactivated fetal bovine serum (FBS), 0.7 mM Glutamine, 50 units/mL penicillin, 50 μg/mL streptomycin) was added and the islets were maintained in culture for up to 35 days under standard conditions. Once a week, half of the total medium was replaced with fresh medium.

Twelve preparations were used; the donor characteristics are: 5 women and 7 men, donor age 52 ± 5 years, BMI 25.8 ± 3.7 kg/m^2^, islets purity 83 ± 6.5%.

### *In vitro* cellular assessments

*Immunofluorescence* was performed as previously described^63^. See supplementary material and methods for a list of antibodies.

#### Cell growth

The cells were fixed at 5, 10, 15 and 25 days after seeding in culture and labeled with anti-insulin (β-cells marker), anti-chromogranin A (endocrine cells marker) antibodies and DAPI. In order to quantify cell growth and differentiation, random fields from each sample were photographed at 40x magnification and the number of chromogranin A- (endocrine cells), insulin- (β-cells) and DAPI- (total cells) immunopositive cells was determined. For each sample, the percentage of endocrine cells over the total number of cells in the field (chromogranin A-positive cells over DAPI-positive cells) and the percentage of β-cells over endocrine cells in the field (insulin-positive cells over chromogranin A-positive cells) were calculated. The mean values and standard deviations were evaluated on the basis of four independent experiments performed in triplicate.

#### TIRFM microscopy and quantitative immunofluorescence

The islet cells were fixed at 15 days of culture and labeled with anti-vinculin antibodies, FITC-phalloidin and DAPI. Random fields were imaged by epifluorescence (actin and DAPI) and TIRF (vinculin) microscopy using a Carl Zeiss TIRF microscope, equipped with an Argon laser, using a 100 × 1.45 numerical aperture (NA) oil immersion objective. Green fluorescence was excited using the 488-nm laser line and imaged with a band-pass filter (Zeiss) mounted on a Retiga SRV CCD camera. The images were analyzed using an existing Image-Pro Plus plug-in (object analysis) for selecting and quantifying fluorescent clusters according to their shape, size and fluorescence intensity. For each object, the following parameters were measured in a software-assisted manner: the area (μm^2^), major axis (μm), minor axis (μm) and the number of vinculin-positive clusters per cell; the length (μm) of actin fibers; the nuclear area (μm^2^) and aspect (Major axis/minor Axis, where 1.0 corresponds to a perfect circle).

#### Apoptosis

Immunofluorescence assay: In order to evaluate β-cell apoptosis, the islets cultures were fixed at 2, 10 and 20 days and labeled with DeadEnd™ Fluorometric TUNEL System (Promega), anti-insulin antibody and DAPI^41^. Random fields from each sample were photographed at 40x magnification. In order to quantify the percentage of apoptotic cells, the number of TUNEL-positive β- and non β-cells was counted in at least 100 insulin-immunoreactive β-cells and 100 insulin-immunonegative cells (non-β-cells) in blind, by two different observers (AG and CP). Mean values and standard deviations were evaluated on the basis of three different islet isolations and the experiments were performed in triplicate.

#### Insulin secretion

The total insulin content and glucose-stimulated insulin secretion were measured in lysates and media of freshly isolated intact islets (three day-old islets) or islets cultured on different substrates for up to 20 days by means of an ELISA immunoassay (Mercrodia) as previously described^41^. Insulin secretion was measured in static incubations, over a 30 min period at basal 3.3 mM (Basal release) or stimulatory 16.7 mM glucose (Stimulated release) concentrations.

#### Ca^*2*+^ imaging

Changes in intracellular free Ca^2+^ were determined by single cell Fluo3 fluorescence imaging as previously described^41^. The islets were incubated at 37 °C for 40 min with 2 mM Fluo3/acetoxymethyl ester (Fluo3-AM) (Sigma) supplemented with 0.0125% Pluronic F-127 (Sigma) in KRH solution containing 20 mM HEPES, pH 7.4, 2 mM CaCl_2_, 3.3 mM glucose, followed by 10 min incubation at room temperature in KRH solution prior to imaging. The images were acquired every 10 s with a high resolution Retiga SRV CCD camera (QImaging) attached to a Zeiss Axiovert 200 inverted fluorescence microscope. The fluorescence signal of single cells was measured over time using the Image-ProPlus (Media Cybernetics) software program and the data were expressed as relative total fluorescence [F/F_0_: ratio of fluorescence signal in each frame to basal value (F_0_)] as a function of time^41^.

### Shotgun mass spectrometry and label free quantification

The cells were washed with PBS containing one complete mini protease inhibitor cocktail tablet (Roche), 10 nM Calyculin A, 10 nM Microcystin-LR, 1X Phosphatase Inhibitor Cocktail tablet. They were then scratched from the substrates with a cell scraper, pelletted by centrifugation and analyzed by tandem mass specrometry on a LTQ Orbitrap Velos (Thermo Fisher Scientific, Bremen, Germany)^64^.

After reduction and derivatisation, the proteins were digested with trypsin sequence grade trypsin (Roche) for 16 h at 37 °C using a protein:trypsin ratio of 50:1. LC-ESI-MS/MS analysis was performed on a Dionex UltiMate 3000 HPLC System with a PicoFrit ProteoPrep C18 column (200 mm, internal diameter of 75 μm) (New Objective, USA). Gradient: 1% ACN in 0.1% formic acid for 10 min, 1–4% ACN in 0.1% formic acid for 6 min, 4–30% ACN in 0.1% formic acid for 147 min and 30–50% ACN in 0.1% formic for 3 min at a flow rate of 0.3 μL/min. The eluate was electrosprayed into an LTQ Orbitrap Velos (Thermo Fisher Scientific, Bremen, Germany) through a Proxeon nanoelectrospray ion source (Thermo Fisher Scientific). The LTQ-Orbitrap was operated in positive mode in data-dependent acquisition mode in order to automatically alternate between a full scan (m/z 350–2000) in the Orbitrap (at resolution 60000, AGC target 1000000) and subsequent CID MS/MS in the linear ion trap of the 20 most intense peaks from full scan (normalized collision energy of 35%, 10 ms activation). Isolation window: 3 Da, unassigned charge states: rejected, charge state 1: rejected, charge states 2+, 3+, 4+: not rejected; dynamic exclusion enabled (60 s, exclusion list size: 200). Five technical replicate analyses of each sample were performed. Data acquisition was controlled by Xcalibur 2.0 and Tune 2.4 software (Thermo Fisher Scientific).

Mass spectra were analysed using MaxQuant software (version 1.3.0.5). Enzyme specificity was set to trypsin and a maximum of two missed cleavages were allowed. Carbamidomethylcysteine was set as a fixed modification, N-terminal acetylation, methionine oxidation and serine/threonine/tyrosine phosphorylation as variable modifications. The spectra were searched using the Andromeda search engine against the human Uniprot sequence database (release 04.07.2014) and the mouse Uniprot sequence database (release 29.06.2015). The reversed sequences of the target database were used as decoy database. Protein identification required at least one unique or razor peptide per protein group.

Quantification in MaxQuant was performed using the built-in XIC-based label free quantification (LFQ) algorithm with fast LFQ. The required false positive rate was set to 1% at peptide level and 1% at protein level against a concatenated target decoy database and the minimum required peptide length was set at 6 amino acids. Statistical analyses were performed using Perseus software (version 1.4.0.6, www.biochem.mpg.de/mann/tools/). Only proteins present and quantified in at least 3 out of 4 technical repeats were considered as positively identified in a sample and used for statistical analyses. An ANOVA test (false discovery rate 0.05) was carried out to identify proteins differentially expressed in the three conditions. Common proteins were considered differentially expressed if they were only present in one of the growing conditions or showed significant t-test difference (cut-off at 5% permutation-based False Discovery Rate). These proteins were filtered for further analyses. We filtered out proteins known to be pancreas contaminants by comparing the proteins identified with the high quality islets proteome described in^42,43^.

Proteins differently expressed were clustered according to their functions using the Panther platform^65^ and DAVID software^66^ in order to cluster enriched annotation groups of Molecular Function, Biological Processes, Biological Cellular Component, KEGG pathway, molecular complexes (CORUM) and keywords within the set of identified proteins. Protein-protein interactions were analyzed by Reactome^67^ and String^68^. Functional grouping was based on p-value (≤0.5).

The mass spectrometry proteomics data have been deposited to the ProteomeXchange Consortium via the PRIDE^69^ partner repository with the dataset identifier PXD007569.

### Statistical analysis

Data are presented as means ± S.E. or S.D. of at least three independent experiments. The number of replicates for each experiment is reported. Statistical comparisons were performed using the unpaired Student’s t test or analysis of variance followed by multiple post hoc comparison analyses carried out using Tukey’s test (Prisma). The difference was considered statistically significant when the p value was < 0.05.

### Ethical approval

Human islets were received from Niguarda Ca’ Granda hospital. Islet isolation and islet studies were approved by the Ethics Committee of the Niguarda Ca’ Granda hospital in Milan (11.12.2009). All of the procedures were performed in accordance with the relevant guidelines and national regulations. The Niguarda Ca’ Granda hospital and the Università degli Studi di Milano, waived the need for consent from the donors because islets are only used for experimental research when deemed unsuitable for clinical purposes and would otherwise be destroyed. In cases of this kind obtaining informed consent is not mandatory in Italy.

## Electronic supplementary material

supplementary information

Table S1

Table S2

Table S3

Table S4

Table S5

Table S6
