# Author Correction: Cluster-assembled zirconia substrates promote long-term differentiation and functioning of human islets of Langerhans

**DOI:** 10.1038/s41598-018-35958-4

**Published:** 2018-11-27

**Authors:** Alessandra Galli, Elisa Maffioli, Elisa Sogne, Stefania Moretti, Eliana Sara Di Cairano, Armando Negri, Simona Nonnis, Giuseppe Danilo Norata, Fabrizia Bonacina, Francesca Borghi, Alessandro Podestà, Federico Bertuzzi, Paolo Milani, Cristina Lenardi, Gabriella Tedeschi, Carla Perego

**Affiliations:** 1 Dipartimento di Scienze Farmacologiche e Biomolecolari, Università degli Studi di Milano, via Trentacoste 2, 20134 Milan, Italy; 2 Dipartimento di Medicina Veterinaria, Università degli Studi di Milano, via Celoria 10, 20133 Milan, Italy; 3 Fondazione Filarete, v.le Ortles 22/4, 20139 Milan, Italy; 4 CIMAINA and Dipartimento di Fisica, Università degli Studi di Milano, via Celoria 16, 20133 Milan, Italy; 5 Niguarda Hospital, Milan, Italy; 6 Present Address: Biological and Environmental Science and Engineering Division, KAUST, Jeddah, Saudi Arabia

Correction to: *Scientific Reports* 10.1038/s41598-018-28019-3, published online 02 July 2018

In this Article, Figure 4B panel f) is a duplication of panel e). The correct Figure 4 is shown below as Figure 1.

**Figure 1:**
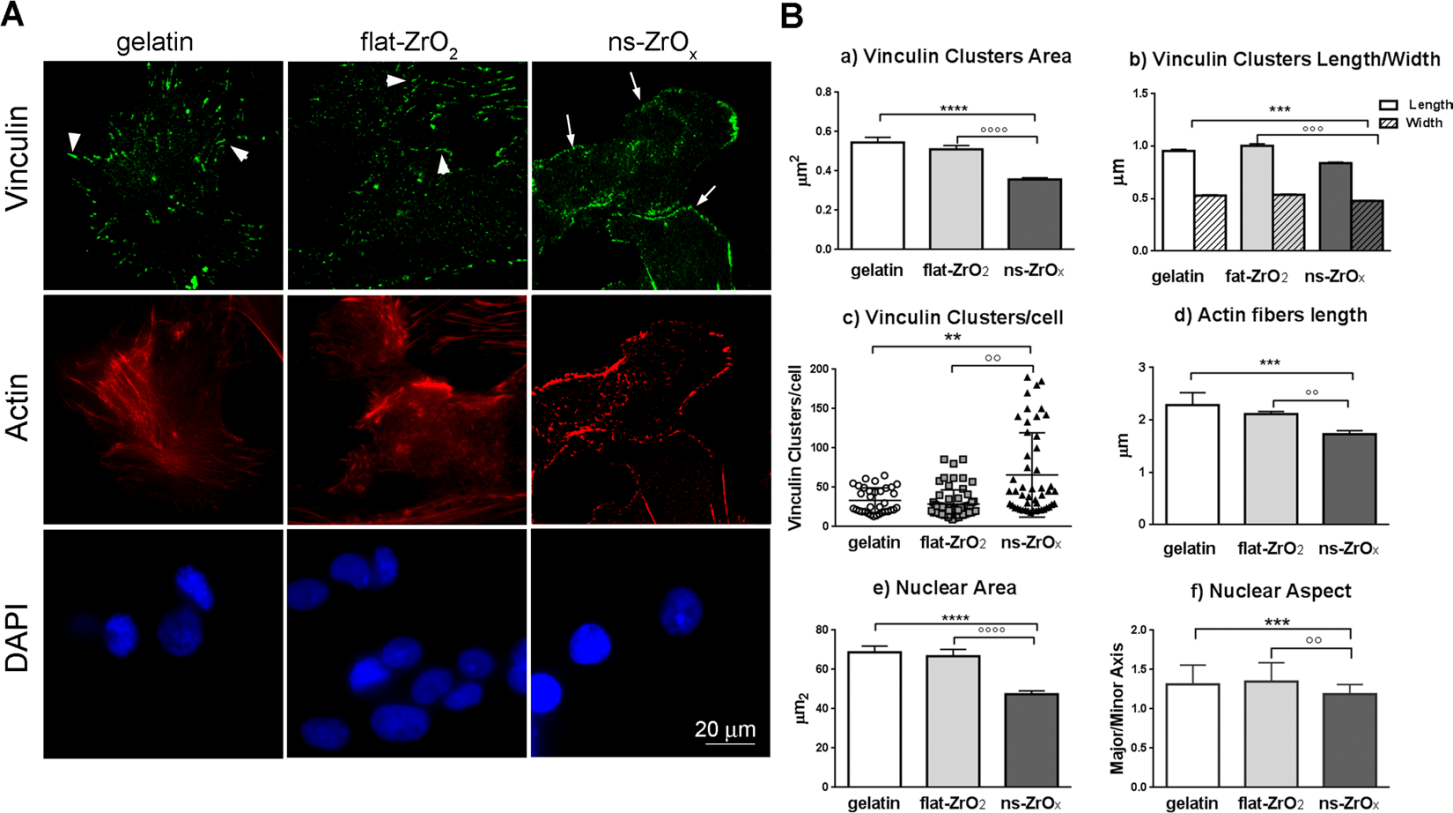
Nanostructured zirconia substrates promote the activation of a mechanotransduction pathway. (**A**) Cells, grown on different substrates for 15 days, were triple stained with anti-vinculin antibody (green), phalloidin (actin, red) and DAPI (blue). Representative epifluorescence (actin and DAPI) and TIRFM (vinculin) images are shown. Bar: 20 μm. Arrows indicate focal complexes, arrowheads indicate focal adhesion. (**B**) Quantitative analyses of adhesive complexes, actin fibers organization and nuclear architecture of cells grown on different substrates. (a,b) Vinculin-positive clusters area, length and width; (c) number of vinculin clusters per cell; (d) cytoskeletal actin fibers length; (e,f) nuclear area and aspect (major/minor axis). Bars illustrate the average responses ± SE (N = 40–100 cells for each substrate) in two different islet preparations. (***p < 0.005, ns-ZrOx vs gelatin; °°p < 0.01, °°°p < 0.005, ns-ZrO_x_ *vs* flat-ZrO_2_).

