# Supplementary material for: Cluster-assembled zirconia substrates promote long-term differentiation and functioning of human islets of Langerhans
Source: Sci Rep. 2018 Jul 2;8:9979. doi: 10.1038/s41598-018-28019-3 (PMC6028636; doi:10.1038/s41598-018-28019-3)
Supplement: Supplementary file 1 — supplementary information [file 41598_2018_28019_MOESM1_ESM.pdf]

Title: Cluster-assembled zirconia substrates promote long term differentiation and functioning of human islets of Langerhans

Author Affiliation:

Alessandra Galli[1]*, Elisa Maffioli[2,3]*, Elisa Sogne[3,4°], Stefania Moretti[1], Eliana di Cairano[1], Armando Negri[2,3], Simona Nonnis[2,3], Giuseppe Danilo Norata[1], Fabrizia Bonacina[1], Francesca Borghi[4], Alessandro Podestà[4], Federico Bertuzzi[5], Paolo Milani[4], Cristina Lenardi[3,4], Gabriella Tedeschi[2,3], Carla Perego[1]#

SUPPLEMENTARY FIGURES, TABLES and MATERIAL AND METHODS

Key words:

Islet of Langerhans, Insulin, diabetes, Cell-matrix Interactions, Nanomaterials, Zirconia, Shotgun proteomic

SUPPLEMENTARY MATERIAL AND METHODS

Immunofluorescence was performed as previously described (*11*). The following primary antibodies were used: guinea pig anti-insulin (Dako A0564) 1:1000, mouse anti-chromogranin A (BioGenex, Clone LK2H10) 1:300, rabbit anti-glucagon (Dako) 1:500, mouse anti-Ki67 1:150 (Dako) rat anti-somatostatin (Chemicon, clone YC7) 1:100, mouse anti-vinculin (Sigma) 1:75 and rabbit anti-α smooth muscle actin (Sigma, clone 1A4) 1:200. The following secondary antibodies were used: fluorescein isothiocyanate (FITC)-conjugated anti-mouse and anti-rat IgGs, rhodamine-conjugated anti-mouse and anti-guinea pig IgGs and cyanine CY5-conjugated anti-rabbit IgGs. All secondary reagents were from Jackson ImmunoResearch Laboratories, West Grove, PA, and were used at 1:300. At the end of the secondary antibody incubation, cells were stained with 4',6-diamidino-2-phenylindole (DAPI) 1:10,000 and rhodamine-conjugated phalloidin 1:500 for 20 minutes to label the nuclei and the actin cytoskeleton, respectively.

Necrosis. Islet cells were cultured for 20 days onto different substrates and necrosis was quantified with the LDH Cytotoxicity Assay Kit (Pierce Thermo Fisher Scientific) following the manufacturer's protocols. Data were expressed as percentage of positive controls.

Cell proliferation. *Immunofluorescence.* To evaluate β-cell proliferation, islets cultures were fixed at 5 and 15 days and triple stained with anti-Ki67 (as a marker of cell proliferation), anti-insulin antibodies and DAPI. Random fields from each sample were photographed at 100x magnification. To quantify the percentage of proliferative cells the number of Ki67-positive β- and non-β-cells was evaluated in at least 100 insulin-immunoreactive β-cells and 100 insulin-immunonegative cells (non-β-cells), in blind, by two different observers (AG and CP). Mean values and standard deviations were evaluated on the basis of three independent experiments performed in duplicate.

FACS analysis. Human islets grown on different substrates for 5 or 10 days were dissociated in PBS 5% FCS with 1 mg/mL of collagenase II (SIGMA, Cat#C6885) and 5 mM $CaCl_2$ for 30 minutes at 37°C under agitation. Collagenase was stopped by adding MACS buffer (PBS 2% FCS, 2 mM EDTA). Dissociated islets were fixed and permeabilised (BD Cytofix/Cytoperm, Cat#555028), stained with primary anti-human insulin (DAKO) for 30 minutes at 4°C, washed and stained with secondary PE conjugated anti-guinea pig and ef450 conjugated anti-human Ki67 (eBioscience, Cat#48-5699-41) for 30 minutes at 4°C. Cells were acquired with Novocyte flow cytometer and analysed with Novoexpress software.

Western blotting. Islet cells were collected and solubilized in lysis buffer (20 mmol/L Tris-HCl, pH 7.4, 150 mmol/L NaCl, 1% Triton X-100, Complete mini protease inhibitor (Roche)) for 45 minutes

at 4°C. Protein concentration was determined by BCA assay (Pierce) and equal amounts of total protein (15 µg) were resolved by 10 % SDS-PAGE and transferred onto nitrocellulose membranes (Millipore). The primary antibodies were applied for 2 hrs at 4 °C in blocking buffer (20 mM Tris, pH 7.4, 150 mM NaCl, 0.1% Tween 20 and 5% non-fat dry milk). The following primary antibodies were used: mouse anti-βactin (NovusBio, NB600-501), rabbit anti-β-catenin (Sigma, C2206), mouse anti-αSMA (Sigma, 1A4), rabbit anti-HIF1α (Sigma, SAB2101039), mouse anti-IκBα (Cell Signaling, L35A5), rabbit anti-Phospho-IκBα (Ser32) (Cell Signaling, 14D4), rabbit anti-Phospho-NFκB p65 (Ser536) (Cell Signaling, 93H1), Rabbit anti-NFκB p65 (Cell Signaling, D14E12 XP®). The secondary antibodies HRP-conjugated anti rabbit or anti mouse (Cell Signaling) were used at 1:1000 dilutions. For NFκB and IκBα, the membranes were first stained with the antibody against the phosphorylated forms of the proteins, stripped (2 times incubation at room temperature for 10 min in 0.1 mol/L glycine-HCl, pH 2.2, 0.1% Tween 20; followed by 4 washes in TBS buffer) and re-probed with the anti-protein antibodies. Proteins were detected using the ECL prime detection system (GE Healthcare) taking advantage of Odyssey Fc Imaging system (Li-Cor, Biosciences). Band intensity was quantified by Image Studio™ Lite software (Li-Cor, Biosciences).

SUPPLEMENTARY FIGURES

Figure S1. Nanostructured Zirconia substrates preserve the endocrine cell organization of native islets in long term cultures. **A.** AFM topographic map (top view) of gelatin-coated glass coverslip (vertical scale: 10 nm, rms roughness less than 1 nm). Typical thickness of the proteins in the gelatin layer: 1-2 nm. The films appear granular at the nanometric scale. **B.** Representative light microscopy images of human islets grown on gelatin, flat-ZrO_2 and 15-ns-ZrO_x or 20-ns-ZrO_x for 15 days. Bar: 60 µm. **C.** Total protein content in islets grown on control, flat or nanostructured substrates (15, 25 nm) for 20 days. Data are the mean ± SE of three independent experiments performed in triplicate. *$p<0.05$. **D.** Representative confocal images of human islets grown on different substrates for 5 and 20 days and triple stained with insulin (red), glucagon (green) and somatostatin (blue). Bar=20 µm. **E.** Representative confocal images of human islets grown on different substrates for 20 days and triple stained with anti-insulin (red), anti α-Smooth Muscle Actin (α-SMA) (marker of myofibroblast cells) (green) and DAPI (blue). Bar=10 µm.

Figure S2. Work Flow of quantitative proteomic analyses of cells grown on ns-ZrO_x, gelatin and flat-ZrO_2. Islets from cells grown on different substrates for 20 days were collected, lysed and processed by a shotgun proteomic approach using label free for quantitation. Protein identification required at least one unique or razor peptide per protein group, FDR 0.01 and the minimum peptide length of 6 amino acids. Proteins differentially expressed among the three conditions were identified by an ANOVA test (FDR 0.05). The comparison between specific data sets was carried out by Welch's t-test (p value 0.0167). Common proteins were considered differentially expressed if they were present only in one of the growing condition or showed significant t-test difference.

Figure S3: Quantitative proteomic analyses of cells grown on ns-ZrO_x, gelatin and flat-ZrO_2. A. Venn diagram showing the results of quantitative proteomic analyses. The numbers indicate the number of genes only expressed and common among ns-ZrO_x, gelatin and flat-ZrO_2. B. Gene Ontology classification of proteins common and differentially expressed in cells grown on different substrates. The proteins common to all conditions and differentially expressed (Anova test FDR 0.05) (see Table 1) were classified into different biological processes according to the Gene Ontology classification system GO-biological process (GOBP), GO-cellular component (GOCC), GO-molecular function (GOMF) using Panther software. Functional grouping was based on p value ≤0.05. The numbers in the bars indicate the number of genes for each category.

Figure S4: Proteins differentially expressed in ns-ZrO_x, gelatin and flat-ZrO_2. Vulcano plots of proteins differentially expressed in ns-ZrO_x versus gelatin (S4A), ns-ZrO_x versus flat-ZrO_2 (S4B) and flat-ZrO_2 vs gelatin (S4C). The list of proteins is reported in Tables S1-S2 (ns-ZrO_x versus gelatin) Tables S3-S4 (ns-ZrO_x versus flat-ZrO_2) and Tables S5-S6 (flat-ZrO_2 vs gelatin). Proteins whose difference is statistically significant (Welch's t-test p value 0.0167) are reported in GREEN (increased expression) or RED (decreased expression). In GREY the proteins whose difference is not statistically significant.

Figure S5: β-cell death in islets grown on different substrates. Quantification of cell necrosis in human islets grown on the different substrates for 20 days. Data (mean values ± SD) are expressed as percentage of positive control (100% death cells). (n=3, in triplicate)(*$P<0.05$ ns-ZrO_x *vs* gelatin).

Figure S6: β-cell proliferation in islets grown on different substrates. A. Representative immunofluorescence images of islets triple stained with anti-Ki67 (as a marker of proliferation, green), anti-insulin (red) antibodies and DAPI (blue). Bar=10 µm. **B.** Quantification of β-cell proliferation in human islets

grown on different substrates for 5 and 15 days. **(a)** β-cell proliferation is expressed as the percentage of Ki67-insulin-double positive cells over total insulin-positive cells; **(b)** non β-cell proliferation is expressed as the percentage of Ki67-positive cells over total DAPI-positive and insulin-negative cells. The experiment was performed in triplicate, with three different islet preparations. A minimum of 100 cells for islet preparation was counted. **C-E.** FACS analysis of β-cell proliferation. **C.** Representative contour plot of insulin staining of human islets. Islets were dissociated with collagenase and stained with secondary PE conjugated anti-guinea pig, as negative control, or with primary anti-human insulin followed by secondary PE conjugated anti-guinea pig for beta cells identification. **D**. Median intensity of fluorescence for ef450 conjugated anti-human Ki67 of insulin-positive cells detected 5 or 10 days after human islets culture; **E.** representative histograms from one experiment are shown.

SUPPLEMENTARY TABLES

TABLE S1: Proteins differently expressed comparing ns-ZrO_x and gelatin. List of the proteins increased or exclusively expressed on cells grown on ns-ZrO_x annotated with Perseus software. Categorical annotation was supplied in form of Gene Ontology (GO) biological process (BP), molecular function (MF), cellular component (CC), keywords as well as in participation in a KEGG pathway and molecular complexes (CORUM).

TABLE S2: Proteins differently expressed comparing ns-ZrO_x and gelatin. List of the proteins decreased on cells grown on ns-ZrO_x or exclusively expressed in cell grown on gelatin, annotated with Perseus software. Categorical annotation was supplied in form of Gene Ontology (GO) biological process (BP), molecular function (MF), cellular component (CC), keywords as well as in participation in a KEGG pathway and molecular complexes (CORUM).

TABLE S3: Proteins differently expressed comparing ns-ZrO_x and flat-ZrO_2. List of the proteins increased or exclusively expressed on cells grown on ns-ZrO_x annotated with Perseus software. Categorical annotation was supplied in form of Gene Ontology (GO) biological process (BP), molecular function (MF), cellular component (CC), keywords as well as in participation in a KEGG pathway and molecular complexes (CORUM).

TABLE S4: Proteins differently expressed comparing ns-ZrO_x and flat-ZrO_2. List of the proteins decreased on cells grown on ns-ZrO_x or exclusively expressed in cell grown on flat-ZrO_2 annotated with Perseus software. Categorical annotation was supplied in form of Gene Ontology (GO) biological process (BP), molecular function (MF), cellular component (CC), keywords as well as in participation in a KEGG pathway and molecular complexes (CORUM).

TABLE S5: Proteins differently expressed comparing flat-ZrO_2 and gelatin. List of the proteins increased or exclusively expressed on cells grown on flat-ZrO_2 annotated with Perseus software. Categorical annotation was supplied in form of Gene Ontology (GO) biological process (BP), molecular function (MF), cellular component (CC), keywords as well as in participation in a KEGG pathway and molecular complexes (CORUM).

TABLE S6: Proteins differently expressed comparing flat-ZrO_2 and gelatin. List of the proteins decreased on cells grown on flat-ZrO_2 or exclusively expressed in cell grown on gelatin annotated with Perseus software. Categorical annotation was supplied in form of Gene Ontology (GO) biological process (BP), molecular function (MF), cellular component (CC), keywords as well as in participation in a KEGG pathway and molecular complexes (CORUM).

SUPPLEMENTARY FIGURES

FIGURE S1

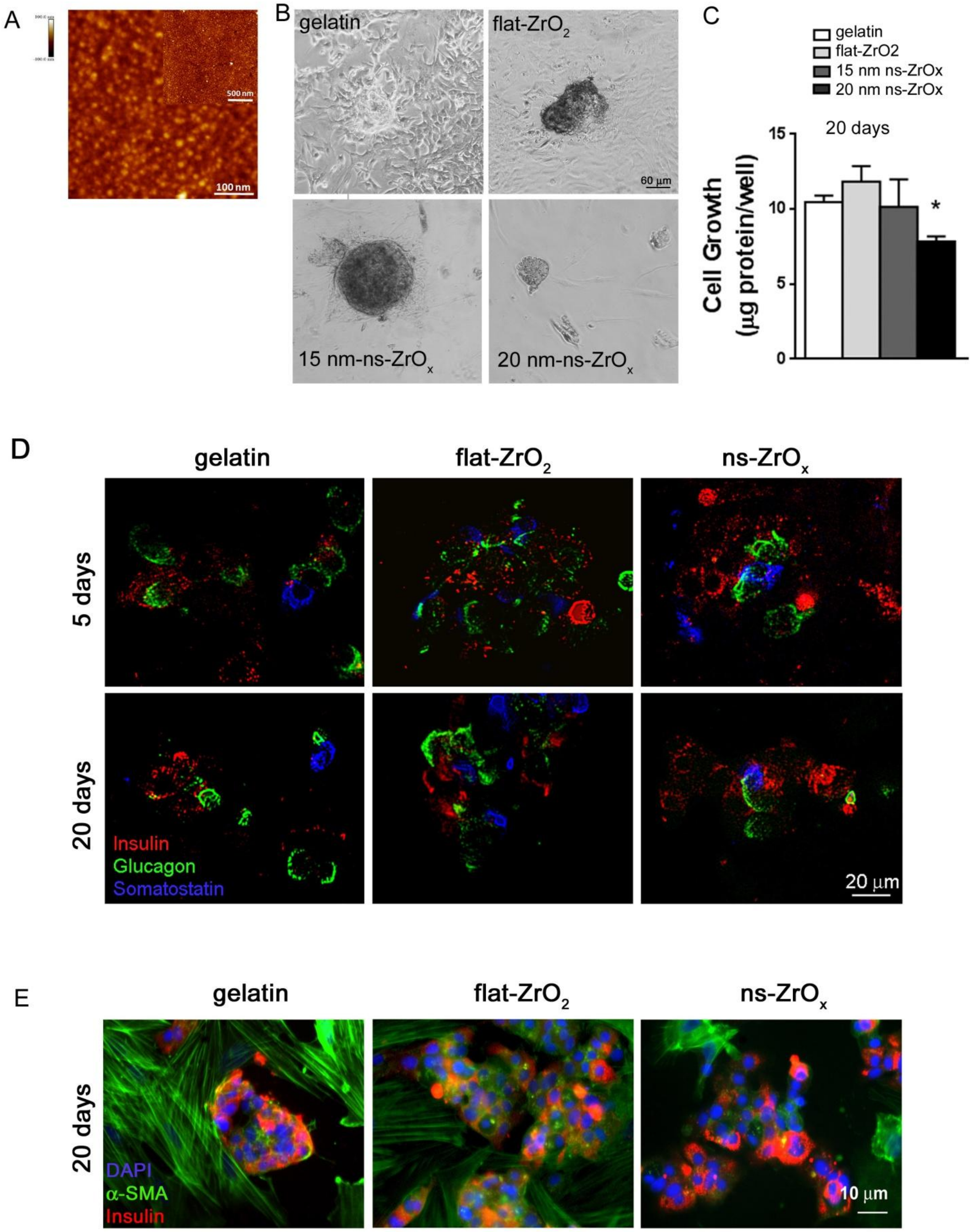

FIGURE S2

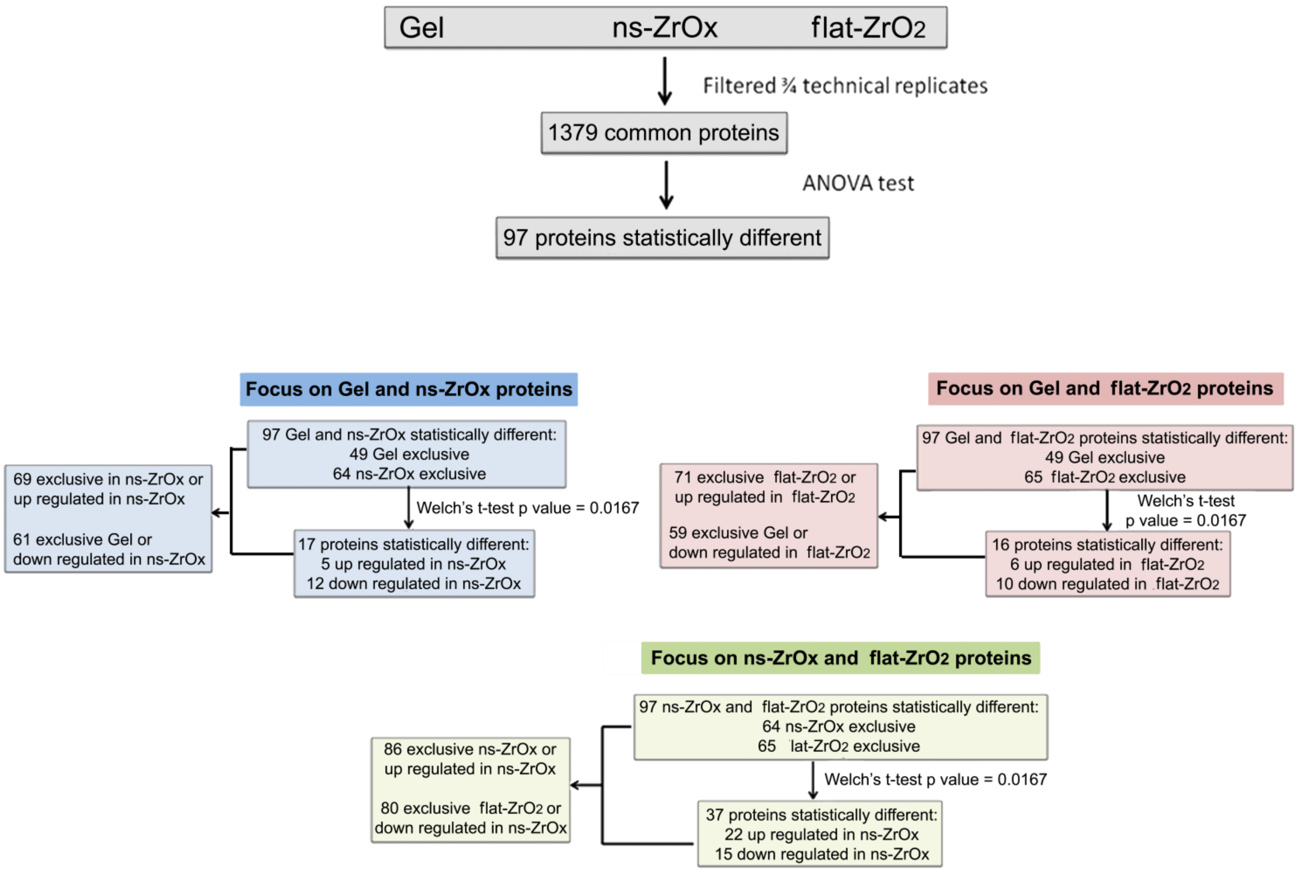

Gel ns-ZrOx flat-ZrO2
Filtered ¾ technical replicates
1379 common proteins
ANOVA test
97 proteins statistically different
Focus on Gel and ns-ZrOx proteins
97 Gel and ns-ZrOx statistically different: 49 Gel exclusive 64 ns-ZrOx exclusive
Welch's t-test p value = 0.0167
17 proteins statistically different: 5 up regulated in ns-ZrOx 12 down regulated in ns-ZrOx
69 exclusive in ns-ZrOx or up regulated in ns-ZrOx
61 exclusive Gel or down regulated in ns-ZrOx
Focus on Gel and flat-ZrO2 proteins
97 Gel and flat-ZrO2 proteins statistically different: 49 Gel exclusive 65 flat-ZrO2 exclusive
Welch's t-test p value = 0.0167
16 proteins statistically different: 6 up regulated in flat-ZrO2 10 down regulated in flat-ZrO2
71 exclusive flat-ZrO2 or up regulated in flat-ZrO2
59 exclusive Gel or down regulated in flat-ZrO2
Focus on ns-ZrOx and flat-ZrO2 proteins
97 ns-ZrOx and flat-ZrO2 proteins statistically different: 64 ns-ZrOx exclusive 65 lat-ZrO2 exclusive
Welch's t-test p value = 0.0167
37 proteins statistically different: 22 up regulated in ns-ZrOx 15 down regulated in ns-ZrOx
86 exclusive ns-ZrOx or up regulated in ns-ZrOx
80 exclusive flat-ZrO2 or down regulated in ns-ZrOx

FIGURE S3

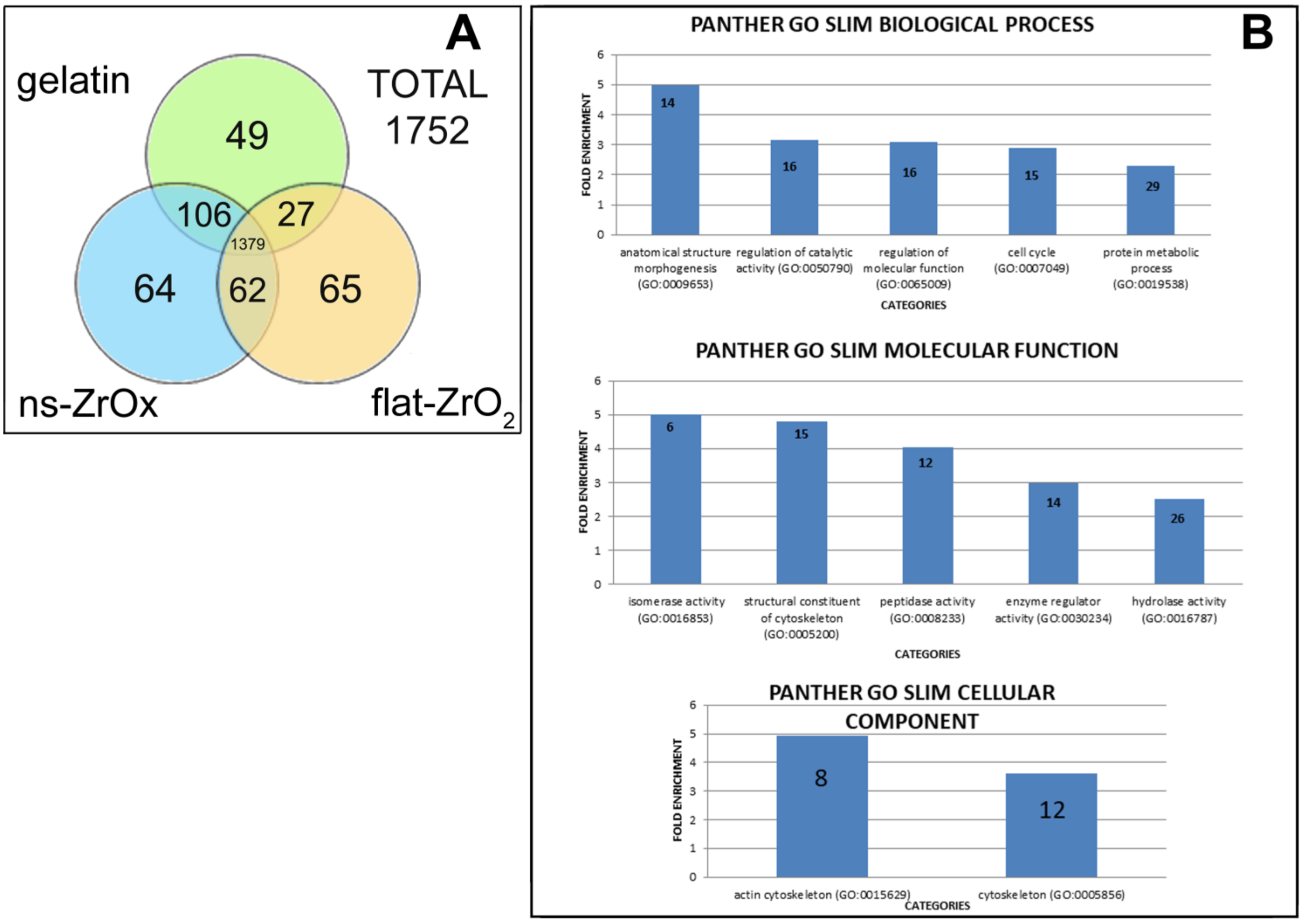
A
gelatin
TOTAL
1752
49
106
27
1379
64
62
65
ns-ZrOx
flat-ZrO2
B
PANTHER GO SLIM BIOLOGICAL PROCESS
FOLD ENRICHMENT
14
16
16
15
29
anatomical structure morphogenesis (GO:0009653)
regulation of catalytic activity (GO:0050790)
regulation of molecular function (GO:0065009)
cell cycle (GO:0007049)
protein metabolic process (GO:0019538)
CATEGORIES
PANTHER GO SLIM MOLECULAR FUNCTION
FOLD ENRICHMENT
6
15
12
14
26
isomerase activity (GO:0016853)
structural constituent of cytoskeleton (GO:0005200)
peptidase activity (GO:0008233)
enzyme regulator activity (GO:0030234)
hydrolase activity (GO:0016787)
CATEGORIES
PANTHER GO SLIM CELLULAR COMPONENT
FOLD ENRICHMENT
8
12
actin cytoskeleton (GO:0015629)
cytoskeleton (GO:0005856)
CATEGORIES

FIGURE S4

A

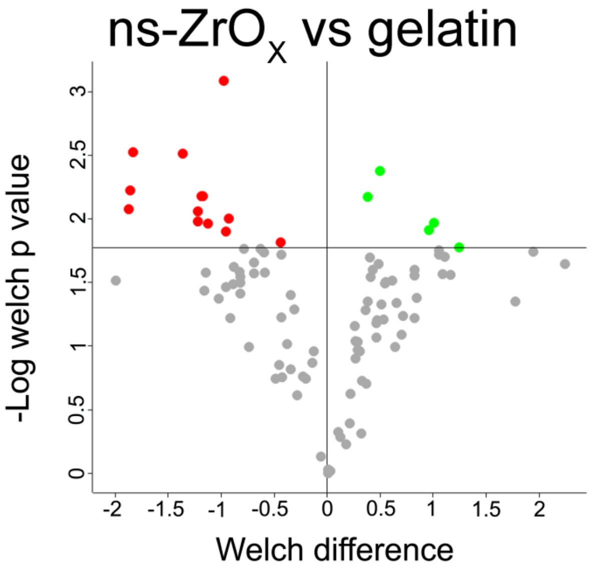

B

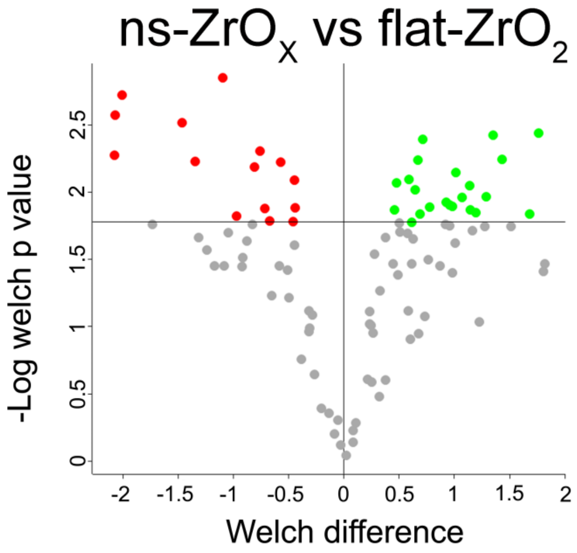

C

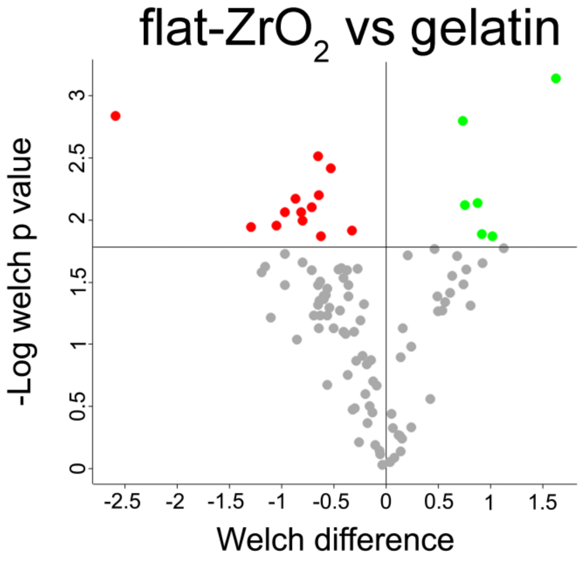

FIGURE S5

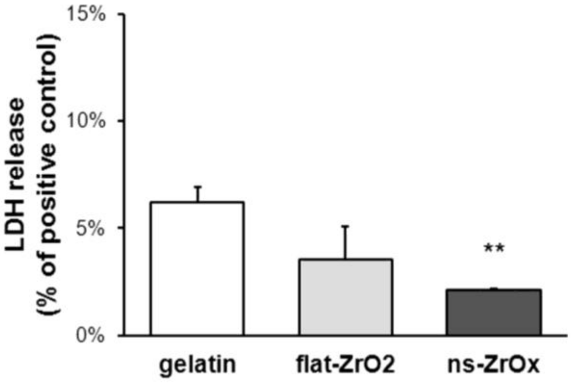
15%
10%
5%
0%
LDH release
(% of positive control)
**
gelatin
flat-ZrO2
ns-ZrOx

FIGURE S6

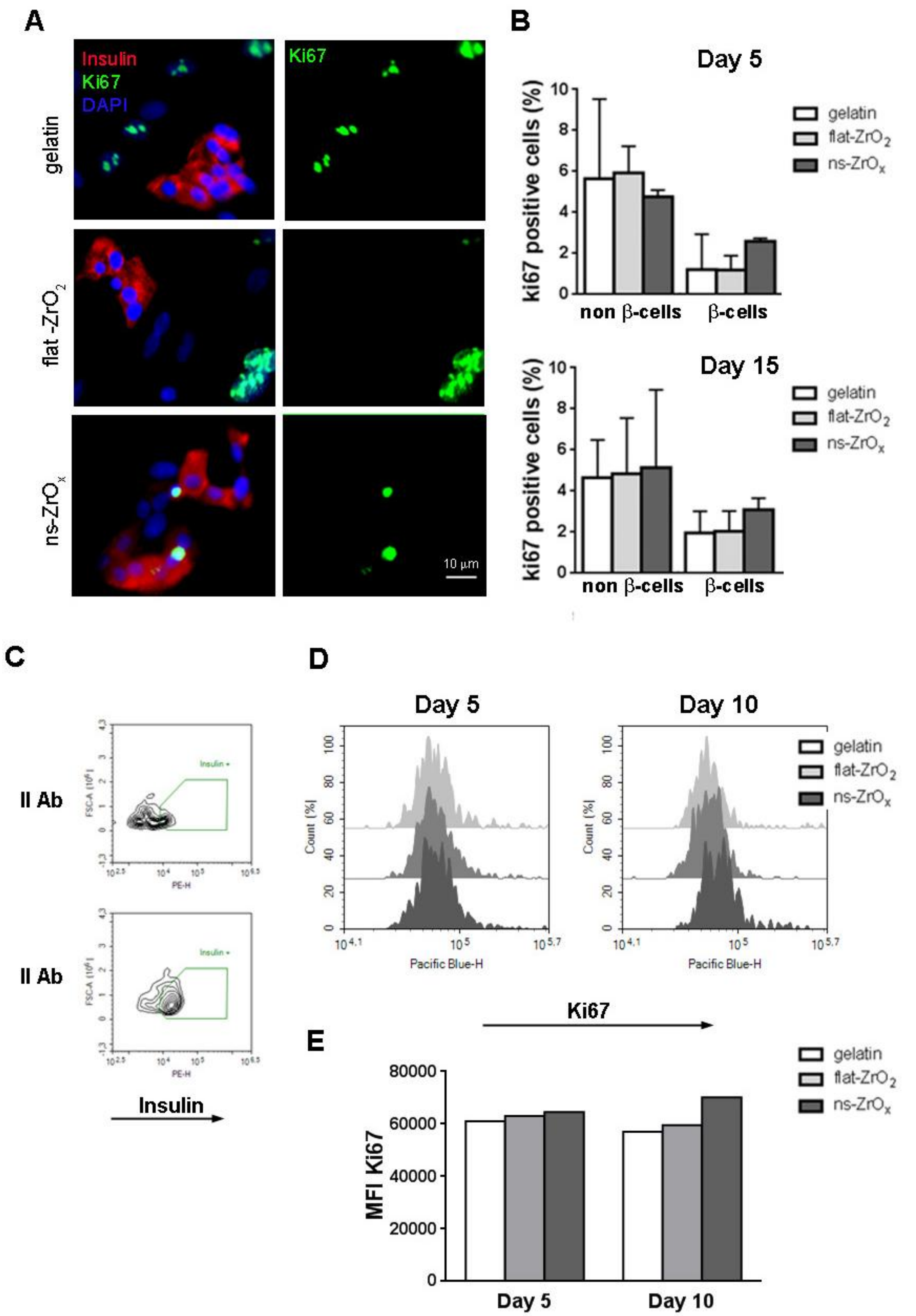
A
Insulin
Ki67
DAPI
Ki67
gelatin
flat -ZrO2
ns-ZrOx
10 µm
B
Day 5
ki67 positive cells (%)
non β-cells
β-cells
gelatin
flat-ZrO2
ns-ZrOx
Day 15
C
II Ab
Insulin +
FSC-A
PE-H
Insulin
D
Day 5
Day 10
Count [%]
Pacific Blue-H
Ki67
E
MFI Ki67
80000
60000
40000
20000
0
Day 5
Day 10
